# Complexation of histone deacetylase inhibitor belinostat to Cu(II) prevents premature metabolic inactivation *in vitro* and demonstrates potent anti-cancer activity *in vitro* and *ex vivo* in colon cancer

**DOI:** 10.1007/s13402-023-00882-x

**Published:** 2023-11-07

**Authors:** Ellen Finnegan, Wei Ding, Ziga Ude, Sara Terer, Tadhg McGivern, Anna M. Blümel, Grainne Kirwan, Xinxin Shao, Flavia Genua, Xiaofei Yin, Alexander Kel, Sarinj Fattah, Parvathi A. Myer, Sally-Ann Cryan, Jochen H. M. Prehn, Darran P. O’Connor, Lorraine Brennan, Gregory Yochum, Celine J. Marmion, Sudipto Das

**Affiliations:** 1 School of Pharmacy and Biomolecular Sciences, RCSI University of Medicine and Health Sciences, Dublin, Ireland; 2 Department of Surgery, Division of Colon & Rectal Surgery, Milton S. Hershey Medical Center, The Pennsylvania State University, Hershey, PA 17036 USA; 3 Department of Chemistry, RCSI University of Medicine and Health Sciences, Dublin, Ireland; 4 Department of Physiology and Medical Physics, RCSI University of Medicine and Health Sciences, Dublin, Ireland; 5 UCD School of Agriculture and Food Science, UCD Conway Institute, Belfield, University College Dublin, Dublin, Ireland; 6 GeneXplain GmbH, Wolfenbuettel, Germany; 7 BIOSOFT.RU, LLC, Novosibirsk, Russia; 8 Institute of Chemical Biology and Fundamental Medicine SBRAS, Novosibirsk, Russia; 9 Montefiore Medical Center, Albert Einstein Cancer Center, Bronx, NY USA; 10 Department of Biochemistry & Molecular Biology, College of Medicine, The Pennsylvania State University, Hershey, PA 17036 USA

**Keywords:** Colon Cancer, Epigenetic, Histone Deacetylase Inhibitor, Copper Complex, Organoids

## Abstract

**Purpose:**

The histone deacetylase inhibitor (HDACi), belinostat, has had limited therapeutic impact in solid tumors, such as colon cancer, due to its poor metabolic stability. Here we evaluated a novel belinostat prodrug, copper-bis-belinostat (Cubisbel), *in vitro* and *ex vivo*, designed to overcome the pharmacokinetic challenges of belinostat.

**Methods:**

The *in vitro* metabolism of each HDACi was evaluated in human liver microsomes (HLMs) using mass spectrometry. Next, the effect of belinostat and Cubisbel on cell growth, HDAC activity, apoptosis and cell cycle was assessed in three colon cancer cell lines. Gene expression alterations induced by both HDACis were determined using RNA-Seq, followed by *in silico* analysis to identify master regulators (MRs) of differentially expressed genes (DEGs). The effect of both HDACis on the viability of colon cancer patient-derived tumor organoids (PDTOs) was also examined.

**Results:**

Belinostat and Cubisbel significantly reduced colon cancer cell growth mediated through HDAC inhibition and apoptosis induction. Interestingly, the *in vitro* half-life of Cubisbel was significantly longer than belinostat. Belinostat and its Cu derivative commonly dysregulated numerous signalling and metabolic pathways while genes downregulated by Cubisbel were potentially controlled by *VEGFA, ERBB2* and *DUSP2* MRs. Treatment of colon cancer PDTOs with the HDACis resulted in a significant reduction in cell viability and downregulation of stem cell and proliferation markers.

**Conclusions:**

Complexation of belinostat to Cu(II) does not alter the HDAC activity of belinostat, but instead significantly enhances its metabolic stability *in vitro* and targets anti-cancer pathways by perturbing key MRs in colon cancer. Complexation of HDACis to a metal ion might improve the efficacy of clinically used HDACis in patients with colon cancer.

**Supplementary Information:**

The online version contains supplementary material available at 10.1007/s13402-023-00882-x.

## Background

Colon cancer is the third most common type of cancer and fourth leading cause of cancer-related deaths worldwide [1]. Despite the advances in treatment modalities and availability of FDA-approved drugs over the last decade, inter-patient responses vary greatly and survival remains particularly low in the metastatic disease setting [2]. As such, new therapeutic alternatives are urgently required to advance treatment outcomes.

Histone deacetylase inhibitors (HDACis) are an emerging class of anti-cancer therapeutics given the involvement of HDAC proteins in cancer initiation and progression [3]. Mounting evidence now suggests that HDACis may be particularly effective in the treatment of colon cancer, where class I and II HDAC overexpression has been frequently reported [4]. In addition, inhibition of HDAC11, the sole class IV HDAC family, has also shown to be sufficient at inducing cell death in the HCT116 colorectal cancer cell line [5].

One HDACi, known as belinostat (PXD101), is a hydroxamate-based pan-HDACi capable of inhibiting class I, II and IV HDAC isoforms. Currently, this HDACi is approved for the treatment of peripheral T-cell lymphoma (PTCL) and has shown promising preclinical results *in vitro* against a variety of human cancer cell lines [6–8]. In addition to hematologic malignancies, various clinical trials for belinostat have also taken place for patients with solid malignancies including colorectal cancer [9, 10]. For instance, it has been reported that combination of belinostat with 5-fluorouracil (5-FU) resulted in disease stabilization in 26% of colon cancer patients with generally well tolerated side effects [11]. Contrastingly, belinostat has shown little activity as a single agent in solid tumor trials, including ovarian cancer where further investigation is needed as it displayed insufficient efficacy [12].

It has been suggested that the modest activity of belinostat in solid tumors is likely attributed to its shortened half-life and underlying pharmacokinetics [13]. To this end, recent reports have subsequently provided evidence that the hydroxamic moiety of belinostat, which is responsible for HDAC binding and inhibition, is susceptible to rapid degradation, predominantly *via* glucuronidation [14]. This reaction is catalyzed by the UGT1A1 liver microsomal enzyme, resulting in extensive metabolism of belinostat and hence inactivation of its cytotoxicity before reaching solid tumor cells [15]. Resulting metabolites of belinostat, such as belinostat glucuronide, have very weak or no cytotoxicity, which may further explain the poor activity of belinostat in solid tumors [16].

To address this issue, complexation of the HDACi to a metal ion *via* its hydroxamate moiety may provide protection from premature degradation and ultimately offer a potential route to enhance the efficacy of belinostat within tumor cells [17]. Hydroxamic acids have a known propensity to bind metal ions. This has been demonstrated with another clinically used HDACi, suberoylanilide hydroxamic acid (SAHA), marketed under the name of vorinostat, which showed a stronger propensity to chelate metal ions such as Cu(II) [18]. Furthermore, previous work from Marmion et al., also demonstrated the added benefit of complexing the hydroxamate-based SAHA to a DNA damaging Cu(II)-phenantholine-type (phen) framework to generate innovative Cu-SAHA-phen derivatives with an enhanced cytotoxicity profile over the parent drug SAHA [17, 19].

Herein, we present the development of a novel belinostat prodrug, known as copper-bis-belinostat or Cubisbel [Cu(Bel_-1H_)_2_], which consists of two belinostat molecules coordinated to one Cu(II) center. We hypothesized that upon entry to the reducing environment of a tumor cell, the redox active-Cu(II) ion would undergo reduction to Cu(I) resulting in the release of two molar equivalents of the active drug, belinostat. Ultimately, by binding two molecules of belinostat to Cu(II) *via* the hydroxamate moiety, rapid metabolic inactivation of belinostat that occurs at this region may subsequently be prevented, thus improving the therapeutic efficacy of belinostat against solid tumors [20].

In this study, we showed that complexation of belinostat to Cu(II) resulted in increased metabolic stability of belinostat *in vitro*. To explore whether the *in vitro* potency of Cubisbel is similar to that of belinostat, and to ensure effective separation of belinostat from the Cu(II) ion in the novel prodrug, we elucidated the cellular mechanisms by which Cubisbel exerts its cytotoxicity in both colon cancer cell lines and patient-derived tumor organoids (PDTOs) and determined that the activity of belinostat is maintained despite its complexation to Cu(II). Importantly, this is also the first report of the transcriptomic alterations induced by both belinostat and Cubisbel in colon cancer cells, some of which suggest a more potent anti-cancer effect of Cubisbel in a cell line-specific manner. Taken together, this comprehensive molecular and phenotypic assessment of belinostat and Cubisbel provides a strong rationale for future investigations using suitable *in vivo* models of colon cancer.

## Methods

### Cell lines and compounds

CACO-2 cells, derived from an early stage colon adenocarcinoma, and paired cell lines SW480 and SW620 (primary tumor and lymph node metastasis pair respectively from the same patient) were purchased from the American Type Culture Collection, ATCC (www.atcc.org), and maintained in a 37°C incubator at 5% CO_2_. SW480 and SW620 cells were cultured in Leibovitz media (L-15) (Sigma Aldrich) supplemented with 10% Fetal Bovine Serum (FBS) (Gibco, Invitrogen), 1% penicillin/streptomycin (Sigma Aldrich) and 1% L-glutamine (Gibco, Invitrogen). CACO-2 cells were cultured in Dulbecco’s Modified Eagle Medium (DMEM) high glucose (Sigma Aldrich) supplemented with 10% FBS, 1% penicillin/streptomycin and 1% MEM Non-essential Amino Acid Solution (Sigma Aldrich).

Copper-bis-belinostat [Cu(Bel_-1H_)_2_] was synthesized in-house as described in the Supplementary Methods. Belinostat glucuronide, the external standard, was purchased from ChemSpace SIA and belinostat was purchased from Sigma Aldrich. All drugs were dissolved in dimethyl sulfoxide (DMSO for cell culture) (Sigma Aldrich).

### Cell viability assay

Cell viability was determined by an MTT (3-(4,5-dimethylthiazol-2-yl)-2,5-diphenyltetrazolium bromide) assay. Cells were seeded in 96-well plates at a density of 1 × 10^4^ cells/well and subsequently exposed to various concentrations of belinostat or Cubisbel for 24, 48 and 72 h. For synergy assays, cells were co-treated with varying concentrations of belinostat + CuCl_2_ ranging from 0.3 µM to 10 µM at a molar ratio ranging from 1:1 to 1:33 for each drug respectively for 72 h. Hence, 15 µl of 5 mg/ml MTT (Sigma Aldrich) prepared in phosphate-buffered saline (PBS) was added to each well and incubated for 3 h at 37°C in darkness. Following removal of MTT and addition of DMSO, absorbency was read on the Perkin Elmer Victor Wallace Multilabel Counter 1420 Microplate Reader at 570 nm. Percentage viability was determined by normalizing the averaged absorbencies of control cells to 100%. Drug IC_50_ values 72 h post-treatment were calculated using GraphPad Prism 5 software (GraphPad Software, Inc., USA v8.3.1) using the log(inhibitor) vs. normalized response—variable slope function.

### *In vitro* clearance of belinostat in HLMs

Incubation of belinostat and Cubisbel with HLMs (150 patient pool) was carried out as previously described by Zhang et al*., *[21]. Briefly, 24 μl UDPGA (UGT Reaction Mix A), 205.5 μl water and 60 μl UGT Reaction Mix B were incubated with 7.5 μl HLMs for 5 min at 37°C. Hence, 3 μl of 10 mM belinostat or Cubisbel was added and incubated for 0, 30, 60 or 90 min at 37°C at a final concentration of 100 nM drug per reaction. Enzymatic reactions were terminated by the addition of 300 μl ice-cold methanol (HPLC-grade). Samples were centrifuged (10,060 × g) for 10 min at 4°C and supernatant analyzed by UPLC-QTOF-MS (see Supplementary Methods). HLMs and UGT reaction mix solutions A and B were purchased from Corning Gentest.

### *In vitro* drug clearance for pharmacokinetic prediction

Belinostat and belinostat glucuronide concentrations (μM) in HLMs, determined using UPLC-QTOF-MS, were subsequently used to plot log % drug remaining versus time, using the 0 min time point as 100%. Slope of the resulting linear regression was used to calculate *in vitro* t_1/2_ (min). The following formula from Obach et al*.,* was used for calculate intrinsic clearance (Cl_int_) at μl/min/mg protein [22]:

$${CL}_{int}\;=\;\frac{(\frac{0.693}{in\;vitro\;{t}_{1/2}})}{(ml\;total\;incubation)\;(mg\;microsome\;protein)}$$

### HDAC activity assay

Nuclear protein extracts were prepared using the EpiQuik Nuclear Extraction Kit I (Epigentek) and concentrations were normalized using a Bicinchroninic acid (BCA) assay. HDAC activity was determined using the EpiQuik HDAC Activity/Inhibition Assay Kit (Colorimetric, Catalog #P-4002), according to the manufacturer’s instructions. In brief, the nuclear protein was incubated with biotinylated HDAC substrate for 1 h. Remaining un-deacetylated substrate was hence captured with a high-affinity antibody and then detected with detection antibody by an enzyme-linked immunosorbent assay (ELISA).

### Cell cycle analysis

Flow cytometry analysis was carried out on cells treated with IC_50_ concentrations of belinostat and Cubisbel. After 72 h, cells were harvested using Trypsin–EDTA (0.05%) (Thermo Fisher Scientific) and washed twice with ice-cold PBS. Cells were fixed in 70% ethanol at 4°C for 48 h and hence treated with 0.1% Triton X-100 and 100 μg/ml RNase A in 1 mL PBS and incubated at 37°C for 10 min. 1 mg/ml PI (propidium iodide) was then added to each sample and analyzed on the BD FACSCanto™ II flow cytometer using BD FACSDIVA™ software. Histograms were generated using FlowJo Software (v10.7.2).

### Apoptosis assay

Apoptosis was detected by flow cytometry using the eBioscience™ Annexin V Apoptosis Detection Kit APC (Catalog no: 88–8007-72) as per the manufacturer’s instructions. Cells were treated with IC_50_ concentrations of belinostat and Cubisbel for 72 h. Hence, adherent cells and cells in media were harvested, washed once in ice-cold PBS and incubated with Annexin V (APC) and PI. Both early apoptotic (Annexin V-positive, PI-negative) and late apoptotic (Annexin V-positive and PI-positive) cells were included in total cell death determinations.

### Reverse transcription and real-time quantitative PCR

RNA was extracted from SW480 and SW620 colon cancer cells treated with CuCl_2_ or DMSO control for 72 h at concentrations equivalent to Cubisbel IC_50_ concentrations using Trizol reagent as described in the manufacturer’s instructions. RNA was converted to cDNA using the High Capacity cDNA Reverse Transcription Kit (Applied Biosystems) and the T100 thermal cycler (Bio-Rad Laboratories, Hercules, CA, USA). Real-time quantitative PCR (RT-qPCR) was carried out using the PowerUp™ SYBR™ Green Master Mix for qPCR (Applied Biosystems) and the 7500 real time PCR system (Applied Biosystems). The 2-ΔΔCT method was used to identify *CD22*, *ZNF114*, *LGALSL*, *GNG2* and *NTRK2* transcript levels relative to the 18S endogenous control, which were normalized to the DMSO vehicle control. Primer sequences for the above genes can be found in Supplementary Table 1.

### RNA sequencing and data analysis

RNA sequencing (RNA-Seq) was carried out on all colon cancer cells treated with IC_50_ concentrations of belinostat and Cubisbel. After 72 h, total RNA was extracted and purified using the Qiagen RNeasy Mini Kit (cat. No. 74104). Library preparation was subsequently carried out in the Genomics Core Technology Unit, Queen’s University Belfast using the Illumina Kappa Hyper RNA library preparation kit followed by RNA sequencing using the Illumina NovaSeq 2000 platform.

Data processing was done using the nf-core RNA-Seq pipline (v3.0, profile: docker) [23] and R (v4.0). In brief, the following steps were carried out: Quality Control (FastQC, v0.11.9) [24], adapter and quality trimming (Trim Galore!, v0.6.6) [25], alignment to GRCh38 (STAR, v2.6.1d) ﻿﻿[26]﻿﻿, quantification (Salmon, v1.4.0) [27] and deduplication (picard, v2.23.9) [28]. Differentially expressed genes (DEGs) were then identified using DeSeq2 (v1.28.0) for each cell line and each drug against DMSO control [29]. Gene ontology was carried out using the GoSeq v3.13. Plots were generated using ggplot2 v3.3.5 [30] and ComplexHeatmap v2.4.2 [31]. Genes ‘uniquely’ regulated by each HDACi were defined as genes significantly up- or downregulated by one drug only while treatment with the second drug corresponded to control levels. Unique drug-specific DEGs were classified as those with a the ratio between Log2(fold change, Cubisbel) and Log2(Fold change, belinostat) > 2 or less than < 0.2.

### Transcription factor binding analysis

Upregulated and downregulated DEGs were subjected to promoter/enhancer analysis using the TRANSFAC® library database on the GeneXplain software platform (release 2020.2, GeneXplain GmbH, Wolfenbüttel, Germany) [32]. The TRANSFAC database consists of composite modules previously described by Kel et al*.,* which are defined as binding motifs present in regulatory regions -1000 bp and + 100 bp relative of each target gene transcriptional start site [33]. Application of the Composite Module Analyst (CMA) method subsequently uses the positional weight matrix (PWM) library in the TRANSFAC database to search for different transcription factor binding sites (TFBS), thus allowing for identification of predicted binding transcription factors (TFs) [34].

### Identification of master regulators

Following identification of TFs, predicted upstream “master regulators” (MRs) of DEGs were subsequently searched for. MRs are defined as genes at the top of the regulatory hierarchy. To this purpose, the upstream analysis approach as described by Kel et al., was applied [35]. In brief, a graph search algorithm was applied to the TRANSPATH signal transduction database to identify MRs of resulting candidate bindings TFs, ultimately building a comprehensive signal transduction network from input DEGs. Total rank scores of each MR was generated by sorting of keynode and CMA score and gene expression data. Lower total ranks indicate greater likelihood of master molecule regulation.

### Patient-derived tumor organoids (PDTOs)

Colon cancer patient-derived tumor organoids (PDTOs) were generated from three colon cancer patients who underwent surgical procedures and consented to having their tissues deposited in the Carlino Family Inflammatory Bowel and Colorectal Disease (IBCRD) biobank at the Pennsylvania State University Milton S. Hershey Medical Center. Clinical information pertaining to these PDTOs can be found in Supplementary Table 2. This study and the IBCRD biobank, were approved by the Pennsylvania State University College of Medicine Institutional Review Board (PRAMSHY98-057, PI: Dr. Walter Koltun, MD).

For colon cancer PDTO culturing, guidelines from Stem Cell Technologies were followed with some modifications. Colon cancer tumor tissue was suspended in 0.5 ml cell dissociation buffer (2 mM EDTA, pH = 8.0; 43.4 mM sucrose; 54.9 mM D-sorbitol dissolved in 1X DPBS) and minced into 0.5 mm^3^ fragments. The mixture was transferred to 10 ml of the same buffer and 0.5 ml of a 100X penicillin/streptomycin antibiotic stock solution (Corning, #30–002-CI), incubated at 40 rpm for 30 min on ice, centrifuged at 300 x g for 5 min and the pellets were then resuspended in 2 ml basal medium (DMEM/F12, Gibco, #11,330–032 supplemented with 1% bovine serum albumen, Gemini, #700-100p). The dissociated tumor tissue was further triturated, filtered and then centrifuged at 300 x g for 5 min. Pellets were resuspended in 50 μl basal media, an equal volume of Matrigel (Corning, Cat^#^ 356231) was added, and 50 μl of the mixture was placed in the center of a well in a 24-well cell culture plate, which was pre-warmed for 2 h at 37 °C. The plates were incubated at 37°C for 20 min to allow the domes to solidify after which 750 μl of human intestinal organoid growth media (Human IntestiCult Organoid Growth Medium, hIOGM, StemCell Technologies, #06010) was added along with 10 μM ROCK inhibitor (Y-27632, Miltenyi, #130–106-138). For PDTO splitting, when PDTOs in a culture dome reached 60–80% confluence, the media was removed, 1 ml of Gentle Cell Dissociation Reagent (GCDR, StemCell Technologies, Cat^#^ 100–0485) was added, the domes were disrupted through gentle pipetting. An additional 1 ml of GCDR was added and the samples were incubated at 40 rpm at room temperature (RT) for 10 min. PDTOs were collected by centrifugation at 300 x g for 5 min, rinsed with 2 ml ice-cold basal medium, and then resuspended in 50 μl of basal medium and an equal volume of Matrigel. 50 μl of the mixture was placed in a well of a pre-warmed 24-well culture dish. Plates were incubated at 37°C for 20 min and then 750 μl of hIOGM was added.

### 3D cell viability assay

Upon treatment with either belinostat or Cubisbel, PDTO viability was evaluated using the CellTiter-Glo 3D Cell Viability Assay kit (Promega, #G9681). Briefly, the reagent was thawed at 4°C overnight, and equilibrated to RT in a 22°C water bath prior to use for 30 min. For assays, the 96-well plates containing PDTOs were equilibrated to RT for approximately 30 min. For each well, 100 µl of reagent was added and the contents were vigorously mixed for 5 min to induce cell lysis. Then, the plates were incubated for an additional 25 min to stabilize the luminescent signal. Finally, luminescence was recorded using a plate reader (Molecular Devices, FlexStation 3) at 1 s/well.

### Immunofluorescence staining of PDTOs

Colon cancer PDTOs were cultured on circular coverslips, and after drug/control treatment they were rinsed with 1X DPBS lacking calcium and magnesium (Corning, #21–031-CV) and fixed with 4% paraformaldehyde (Thermo Fisher, #28,908) at RT for 30 min. The coverslips were washed 3X with 1X DPBS and incubated for 2 h at RT in blocking/staining buffer (5% donkey serum, 0.2% Triton X-100, 1X DPBS). The coverslips were then incubated with the following primary antibodies diluted in blocking/staining buffer overnight at 4°C: anti-Ki67 (1:200, Cell Signaling, #9027 s); anti-E-cadherin (1:400, Cell Signaling, 24E10, #3195 s), and anti-β-Catenin (1:200, BD Transduction Laboratories, # 610154). The next day, coverslips were washed 3X for 10 min and then incubated with the appropriate secondary antibodies (1:500 diluted, Alexa donkey anti-rabbit 568, Invitrogen, #A10042; and Alexa donkey anti-mouse 488, Invitrogen, # A21202) for 2 h at RT. The PDTOs were washed 3X in 1X DPBS, for 10 min each, and stained with 4’,6-diamdino-2-phenylindole (DAPI) (1:500, Thermo Fisher, #62248) in 1X DPBS for 20 min. After three 5 min washes in 1X DPBS, the coverslips were inverted onto a glass slide and mounted with Prolong Anti-Fade Mounting Solution (Invitrogen, #P36982). Images were collected using a Leica SP8 confocal microscope and the sizes of the PDTOs were measured using LAS X imaging software.

### PDTO real-time quantitative PCR

Colon cancer PDTO (21–267 H7) was treated with an IC_50_ dose of either HDACi for 72 h and DMSO served as vehicle control. Total RNA was isolated using a Trizol reagent as described in the manufacturer’s instructions. RNA concentrations were measured using Nanodrop 2000. 1000 ng of RNA was used for cDNA synthesis using an Omniscript RT kit (QIAGEN, #205111). The cDNAs were diluted to 20 ng/ μL and 5 μL was subjected to quantitative PCR using TaqMan probes in a 10 μL reaction using a Gene Expression Master Mix kit (Thermo Fisher, #4369016). TaqMan primer/probe sets were used for the quantitative real-time RT-PCR reactions, performed with a QuantStudio 12K Flex system (Life Technologies). The following Taqman probes were purchased from Thermo Fisher: GAPDH, Hs02758991_g1; BMI1, Hs00180411_m1; PROM1 (CD133), Hs01009259_m1; SMOC2, Hs00405777_m1. Reactions were incubated at 95 °C for 2 min, followed by 60 cycles at 95 °C for 20 s, 60 °C for 20 s and 72 °C for 30 s. Data are presented as relative transcript levels using the 2-ΔΔCT method after normalizing to GAPDH.

### Statistical analysis

DEGs from RNA-Seq data were identified using the DeSeq2 package and Benjamini Hochberg method for correction for multiple comparison. Cut-off points for DEGs included a fold change < 0.5 or > 2 and FDR < 0.05. For all *in vitro* experiments, a student’s t-test was used to generate p-values using GraphPad Prism software v8.3.1 (*, *P* ≤ 0.05; **, *P* ≤ 0.01; ***, *P* ≤ 0.001; ****, *P* ≤ 0.0001) for 3 independent experiments (*n* = 3) and show ± standard deviation (SD).

## Results

### Design and synthesis of a novel belinostat metallo-prodrug, copper-bis-belinostat, which exerts cytotoxicity against colon cancer cells

**Fig. 1:**
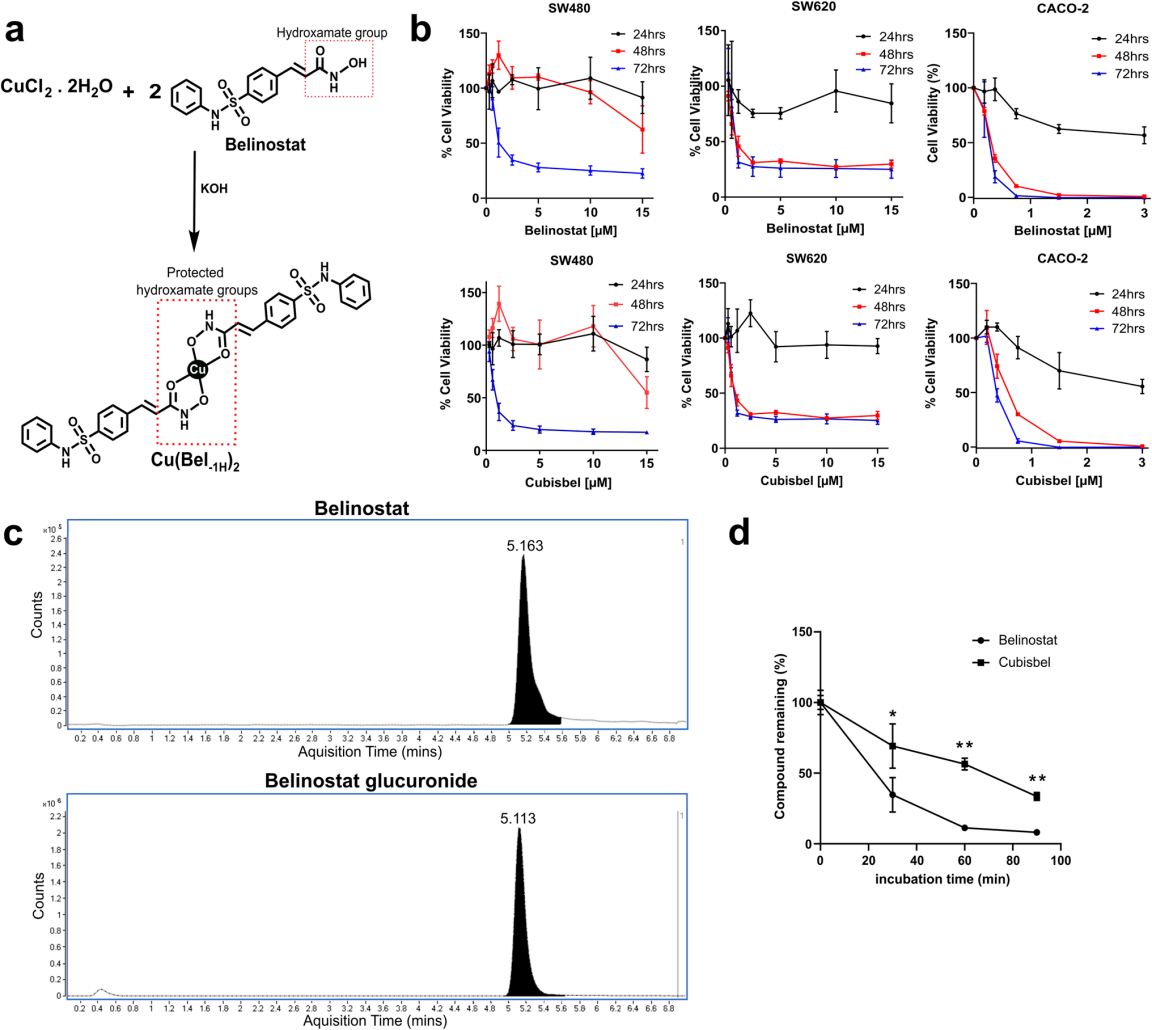
Synthesis and design of copper-bis-belinostat and its effect on colon cancer cell viability and belinostat metabolism in HLMs. **a** Introduction of a copper(II) ion to the hydroxamate moiety of two equimolar concentrations of belinostat afforded formation of novel prodrug copper-bis-belinostat (Cubisbel). **b** An MTT assay was performed on SW480, SW620 and CACO-2 cells after 24, 48 and 72 h of exposure to belinostat and Cubisbel respectively, ranging from 0.1875 µM to 20 µM. Data was plotted against % cell viability and normalized to the DMSO vehicle control. Data is expressed as means for six pooled values from three independent experiments (n = 3) ± SD. **c** Selected extracted ion chromatograms (EIC) of belinostat and belinostat glucuronide from HLM sample. **d** Percentage of belinostat remaining in HLM samples after 0, 30, 60 and 90 min incubation at 37°C with belinostat or Cubisbel HDACis. Points on the plot marked with an asterisk ‘*’ indicate a significant difference of % belinostat drug remaining between Cubisbel and belinostat treatments

Complexation of belinostat to Cu(II) was initiated by treatment of an aqueous solution of CuCl_2_.2H_2_O with the addition of two molar equivalents of a methanolic solution of belinostat in the presence of KOH. The resulting novel complex, namely Cubisbel, was isolated as a green solid in excellent purity, as confirmed by elemental analysis and infrared spectroscopy (Fig. 1a) (see Supplementary Methods). Stability studies of Cubisbel in 100% DMSO were conducted over 10 days using UV–Vis spectroscopy. These studies confirmed that Cubisbel is stable under these conditions (Supplementary Fig. 1).

We first explored the effect of Cubisbel and parent drug belinostat on the viability of SW480, SW620 and CACO-2 colon cancer cell lines respectively (Fig. 1b). Resulting 72 h IC_50_ values were in the low micromolar range, as shown in Table 1. Notably, CACO-2 cells demonstrated a > sixfold and > threefold sensitivity to belinostat and Cubisbel respectively compared to SW480 and SW620 cells. A significant difference between the IC_50_ of Cubisbel and belinostat was uniquely observed in SW480 cells only (*P* = 0.04179). Co-treatment with belinostat and CuCl_2_ at a molar ratio ranging from 1:1 – 1:33 for each drug subsequently indicated evidence of synergy in all cell lines, however, this was predominantly evident in CACO-2 cells (Supplementary Fig. 2) treated with the highest molar ratios of CuCl_2_ compared to belinostat. In summary, Cubisbel exhibited potent cell growth inhibition that may be due to the addition of Cu(II) and is dependent on underlying phenotypes of individual cell lines.

**Table 1:** IC_50_ concentrations of belinostat and Cubisbel in colon cancer cell lines

	Mean IC_50_ (µM ± SD)	
Cell Line	Belinostat	Cubisbel
*SW480*	2.093 ± 0.71	1.131 ± 0.71*
*SW620*	1.416 ± 0.67	1.412 ± 0.26
*CACO-2*	0.263 ± 0.09	0.369 ± 0.02

Using the 72 h time-point of SW480, SW620 and CACO-2 cells treated with belinostat or Cubisbel, IC_50_ concentrations were calculated using Prism (Graphpad v8.3.1). Absorbency values from six wells of three independent experiments were averaged and transformed to the logarithmic values. Hence the non-linear fit function was applied to produce IC_50_ values (log(inhibitor) vs. normalized response—variable slope). Statistical significance was calculated using the student’s t-test (*P* ≤ 0.05), with each data point representing the mean 72 h IC_50_ obtained from n = 3 ± SD. Values marked with an asterisk ‘*’ (*P* ≤ 0.05) indicate a significant difference between belinostat and Cubisbel IC_50_ concentrations

### Rapid metabolism of belinostat is potentially prevented or slowed upon complexation to copper(II) *in vitro*

Rapid phase II metabolism of belinostat *via* glucuronidation, primarily by the UGT1A1 enzyme, is the likely source of the short half-life of this drug, resulting in limited activity in solid tumors such as colon cancer [13]. To first investigate whether the differences between belinostat and Cubisbel IC_50_ concentrations was due to variable UGT1A1 expression in the three colon cancer cell lines, we quantified the protein concentration of this enzyme in each cell line. However, only minimal (< 1 ng/ml) concentrations were identified, which were not significantly different between each cell line (Supplementary Fig. 3). Next, the *in vitro* metabolism of both belinostat and Cubisbel was examined to determine if complexation to Cu(II) may prevent the preliminary degradation of belinostat. As high concentrations of drug metabolizing enzymes, including UGT1A1, which catalyzes this reaction, are present in the liver, UGT1A1-containing human liver microsomes (HLMs) were utilized for investigation of *in vitro* metabolism.

Specifically, for UGT1A1-containing HLMs treated with a final concentration of 100 nM belinostat or Cubisbel, based on concentrations of belinostat used in previous microsomal studies [21], in addition to UGT cofactors, belinostat (*m/z* 317.0583) and belinostat glucuronide (*m/z* 493.0898) were identified at retention time (RT) of 5.16 and 5.11 min, respectively, in line with the external standard (Fig. 1c). Subsequently, the presence of belinostat drug and glucuronide metabolite in the HLMs was validated by tandem mass spectrometry (MS/MS) where a matched MS/MS fragmentation pattern in UGT1A1-containing HLM sample and standard was observed (Supplementary Fig. 4).

After 90 min, marked conversion of belinostat to its glucuronidated product (belinostat glucuronide) was observed following treatment with both drugs, with 8% and 33.5% belinostat remaining from the parent drug and prodrug respectively (Fig. 1d). Based on this timepoint, the estimated *in vitro* t_1/2_ of belinostat and Cubisbel was 23.92 and 59.67 min respectively (Table 2). Importantly, intrinsic clearance of active belinostat was slower (*P* = 0.0041) with Cubisbel treatment compared to belinostat, with a CL_int_ of 57.94 and 23.23 μl/min/mg protein respectively. No conversion to belinostat glucuronide was observed in control microsomes without cofactor addition (Supplementary Fig. 5). Together, these results provide clear evidence that the Cu(II) ion indeed protects the hydroxamate group of belinostat from rapid metabolic inactivation *in vitro*.

**Table 2:** *In vitro* metabolism of belinostat and Cubisbel by UGT1A1-containing human liver microsomes (HLMs)

Compound	t_1/2_ (minutes)	CL_int_ (μl/min/mg protein)
Belinostat	23.92 ± 3.3	57.94 ± 7.3
Cubisbel	59.67 ± 1.6**	23.23 ± 0.6*

Using the 90 min timepoint, the estimated *in vitro* t_1/2_ was calculated using slope of the log % remaining graph. Intrinsic clearance of belinostat (µl/min/mg protein) were hence calculated from *in vitro* t_1/2_ as described in the Methods Section. Each *in vitro* t_1/2_ and CL_int_ value represents mean ± SD from *n* = 3 biological replicates. Statistical significance was calculated using the student’s t-test (*P* ≤ 0.05). Values marked with an asterisk ‘*’ (*P* ≤ 0.05) or ‘**’ (*P* ≤ 0.01) indicate a significant difference between belinostat and Cubisbel t_1/2_ or CL_int_

### Copper-bis-belinostat is a potent inhibitor of HDAC activity that mediates its effect primarily through induction of apoptosis

**Fig. 2:**
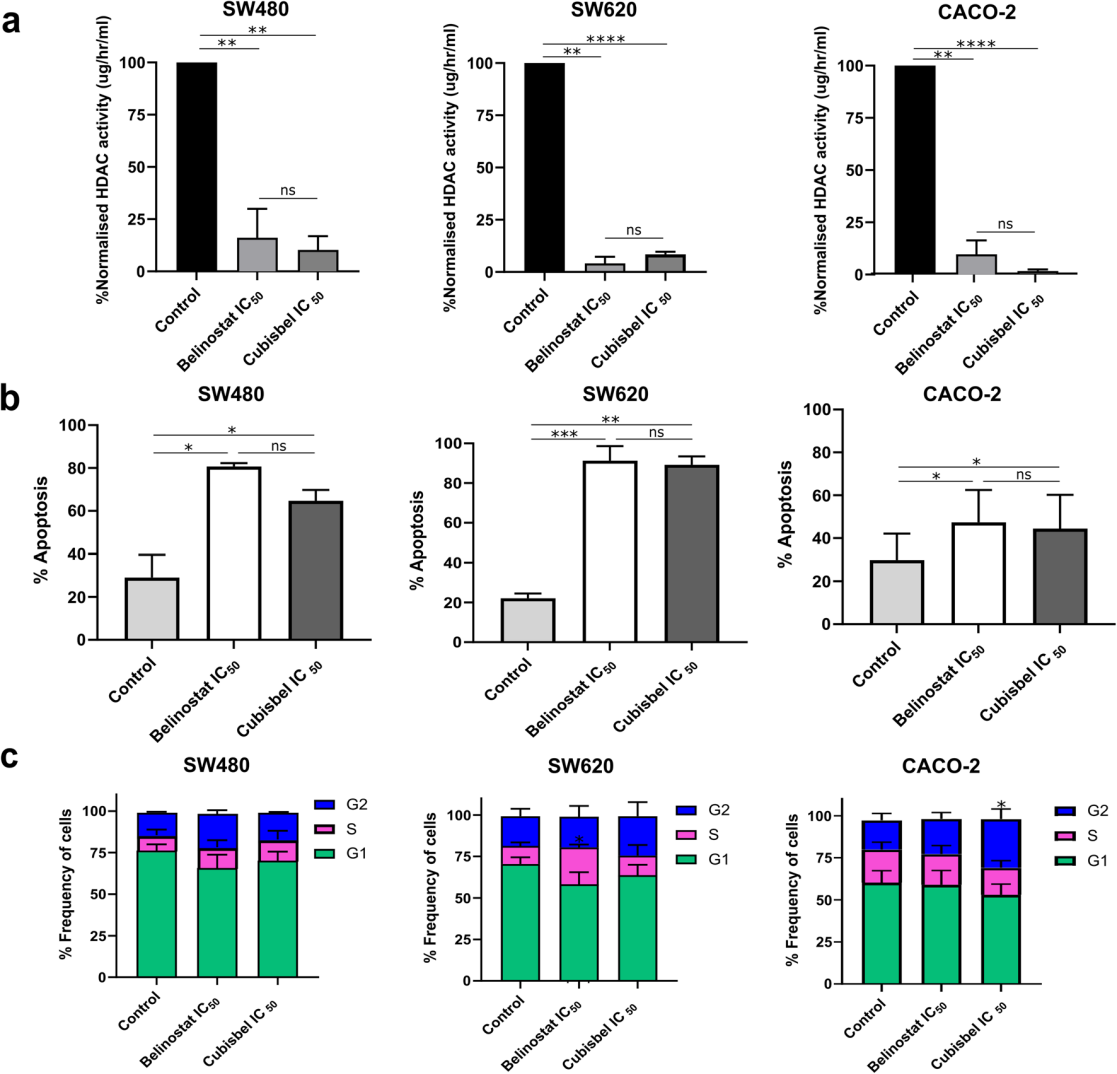
Effect of belinostat and Cubisbel on colon cancer cell HDAC activity, apoptosis and cell cycle. For all phenotypic assays, SW480, SW620 and CACO-2 cells were utilized 72 h post-treatment with the IC_50_ concentrations of belinostat (2.093, 1.416 and 0.263 µM respectively) and Cubisbel (1.131, 1.412 and 0.369 µM respectively). **a** HDAC activity in colon cancer cells following HDACi treatment. Nuclear protein extracts were prepared using the EpiQuik nuclear extraction kit. HDAC activity was measured by the direct colorimetric assay kit EpiQuik (Epigentek). The resulting data was normalized to control cells treated with DMSO only, representing 100% HDAC activity (µg/h/ml). **b** Assessment of apoptosis using Annexin/PI staining following by flow cytometry. The percentage of early and late apoptotic cells (total apoptotic cells) compared to the negative control are illustrated by bar graphs for each cell line. **c** Percentage of cells in each cell cycle phase, graphically represented by stacked columns. The distribution of cells was analyzed by flow cytometry using PI to stain cell DNA. Cell populations in the G0/G1, S and G2/M phase are given as percentages of total cells. For all assays, statistical significance was calculated using the student’s t-test (*P* ≤ 0.05), with each data point representing the mean of *n* = 3 biological replicates ± SD

It is hypothesized that upon entry into the reducing environment of a cancer cell, Cubisbel is activated by the reduction of Cu(II) to Cu(I), allowing for concomitant release of two equivalents of belinostat resulting in potent HDAC inhibition. To this purpose, the effect of belinostat and Cubisbel on overall HDAC activity was next investigated. It was found that both belinostat and Cubisbel significantly inhibited HDAC activity in all three colon cancer cell lines (SW480 *P* = 0.009 and 0.0018; SW620 *P* = 0.0004 and < 0.0001; CACO-2 *P* = 0.0018 and < 0.0001 respectively) (Fig. 2a), with greater significance observed with treatment of Cubisbel.

As interference with HDAC activity can regulate biological processes such as apoptosis and cell cycle in cancer cells [13], we next examined the impact of belinostat and Cubisbel on both processes in colon cancer cells. To that end, we observed a significant increase in total apoptotic cell populations across all colon cancer cells following treatment with belinostat or Cubisbel (SW480 *P* = 0.017 and 0.011; SW620 *P* = 0.0017 and 0.0009; CACO-2 *P* = 0.02 and 0.047 respectively) (Fig. 2b). Moreover, apoptosis was more profound in SW480 and SW620 cells, as demonstrated by the presence of ~ 80% apoptotic cells compared to ~ 50% apoptotic CACO-2 cells treated with belinostat or Cubisbel. In contrast, cell cycle alterations induced by both HDACis were not consistent across the three colon cancer cell lines (Fig. 2c). However, in SW620 cells treated with belinostat, an accumulation of cells in the cell cycle S-phase was observed (*P* = 0.03) and in CACO-2 cells treated with Cubisbel, a G2-phase accumulation of cells was observed (*P* = 0.049). All flow cytometry plots can be found in Supplementary Fig. 6.

To investigate whether any of the phenotypic effects mediated by Cubisbel were due to synergy provided from Cu(II) complexation, HDAC activity, apoptosis and cell cycle activity was additionally examined in the colon cancer cells treated with CuCl_2_ at equivalent doses to that of Cubisbel 72 h IC_50_ (Supplementary Fig. 7). However, no statistically significant impact of CuCl_2_ on any of these phenotypes was observed, suggesting that, at a cellular level, synergy provided by Cu(II) to belinostat was specific to the growth and/or viability of the colon cancer cells.

In summary, these results demonstrated the effective dissociation of Cu(II) from belinostat *in vitro* allowing for subsequent potent HDAC inhibition, which in turn induced significant apoptosis in this colon cancer model.

### Identification of transcriptomic changes by belinostat and copper-bis-belinostat in colon cancer cells

**Fig. 3:**
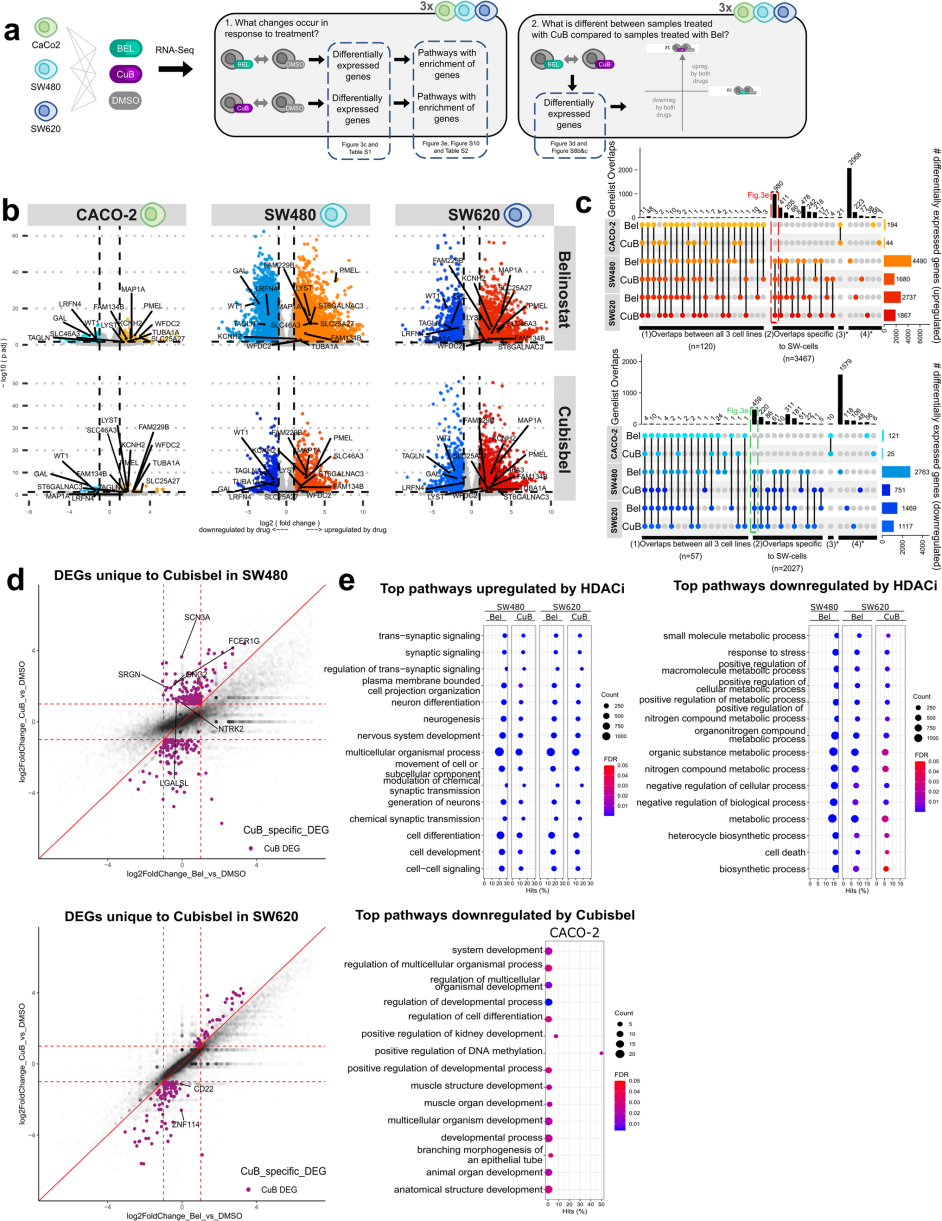
Transcriptomic analysis of genes differentially regulated by belinostat and Cubisbel in colon cancer cells. **a** Overview of workflow for differential gene expression analysis. SW480, SW620 and CACO-2 cells were treated with IC_50_ concentrations of each HDACi and equal concentrations of DMSO control for 72 h and hence subjected to RNA-Seq. **b** Volcano plots of all DEGs (*P* ≤ 0.05) in colon cancer cells treated with belinostat or Cubisbel versus DMSO vehicle control. The x-axis represent log2- fold change plotted against -log10(p-value) on the y-axis. **c** Upset plots showing the number of DEGs upregulated and downregulated by belinostat and Cubisbel in the three colon cancer cell lines versus treatment with DMSO vehicle control. A dotted box represents gene groups used for pathway analysis for subsequent figures. **d** Scatterplots of DEGs significantly dysregulated by belinostat only or Cubisbel only. The x-axis represents the log2foldchange of genes dysregulated by belinostat versus DMSO control, while the y-axis indicates the log2foldchange of genes dysregulated by Cubisbel versus DMSO control. Red dotted lines indicate the cut-off threshold of log2foldchange < 0.5 or > 2. The red diagonal line indicates a slope = 1. Purple dots represent genes up- or downregulated by belinostat only or Cubisbel only, while labelled genes indicate those uniquely regulated by Cubisbel. **e** Dot plots of the top 15 gene ontology (GO) pathways commonly upregulated and downregulated by belinostat and Cubisbel in the SW480-SW620 paired cell line and downregulated by Cubisbel in CACO-2 cells. The dots are sized based on gene count and colored according to their adjusted *P*-value (*P.adj* ≤ 0.05). Significant enrichment of either up- or downregulated genes in pathways was found for all other gene lists with the exception of those downregulated by Cubisbel in SW480 cells and pathways downregulated by belinostat and upregulated by Cubisbel in CACO-2 cells

To date, full transcriptomic alterations induced by belinostat in solid tumor cells have solely been investigated in breast cancer cells, where treatment resulted in enrichment for pathways such as oxidative phosphorylation, cell cycle and *ERBB* signalling [36]. To evaluate the effect of belinostat and the novel Cu-based prodrug on the colon cancer transcriptome, RNA sequencing was carried out following treatment of all three colon cancer cell lines with IC_50_ concentrations of belinostat or Cubisbel (Fig. 3a).

Principal component analysis of colon cancer cells treated with belinostat and Cubisbel demonstrated limited variance between replicates and highlighted the marked contrast between CACO-2 cells and the paired SW480-SW620 cell lines (Supplementary Fig. 8a). As shown in Fig. 3b and c, identification of differentially expressed genes (DEGs) (Supplementary Table 3) indicated a large number of overlapping DEGs between drug treatments in all cell lines. In total, 11 genes were significantly upregulated and 4 genes were downregulated commonly by both HDACi in all three colon cancer cell lines. Moreover, 980 genes were commonly upregulated by both HDACis in SW480 and SW620 cells, while 459 genes were commonly downregulated. Conversely, CACO-2 cells shared minimal DEGs with the paired cell line.

We next examined genes expression changes exclusive to Cubisbel, which remained unchanged with treatment of belinostat (Fig. 3d). Specifically, six genes including *NTRK2* and *LGALSL* were uniquely upregulated by Cubisbel in SW480 cells, while *CD22* and *ZNF114* were uniquely downregulated by Cubisbel in SW620 cells (Supplementary Fig. 8b and c). No genes were uniquely upregulated by Cubisbel in SW620 cells. To determine whether unique regulation by Cubisbel was due to presence of the Cu(II) ion, RT-qPCR analysis of the top genes uniquely regulated by Cubisbel in these cell lines was carried out following treatment with CuCl_2_, however, no significant impact was found (Supplementary Fig. 9).

Following identification of DEGs in the colon cancer cell lines, gene ontology (GO) analysis was subsequently carried out to examine pathways significantly altered by belinostat and Cubisbel (Supplementary Table 4). While no pathways were significantly downregulated by Cubisbel in SW480 cells, the SW480 and SW620 cell lines overall showed multiple similarly dysregulated pathways (Supplementary Fig. 10a and b). We compared pathways enriched across all cell lines and treatments and found multiple overlaps between these two cell lines, however, no overlap with CACO-2 cells was observed. In the SW480-SW620 paired cell line, the top commonly upregulated and downregulated pathway by both HDACis included “*plasma membrane bounded cell projection organization”* and “*small molecule metabolic processes”* respectively (*P* =  < 0.0001). In comparison, “*regulation of developmental process”* and “*multicellular organismal development”* (*P* = 0.004 and 0.01 respectively) were the top significantly downregulated pathways by Cubisbel in CACO-2 cells, while no pathways significantly downregulated by belinostat were identified (Fig. 3e).

Cumulatively, these results demonstrated that both belinostat and Cubisbel have a profound effect on colon cancer cell gene expression. Importantly, at the transcriptomic level, Cubisbel displayed inherent similarities with belinostat, indicating complexation with copper did not negatively impact the potent genome-wide effects of the HDACi [37].

### Upstream analysis reveals genes differentially expressed in response to belinostat and copper-bis-belinostat are regulated by key master regulators

It is widely known that gene expression is controlled by a network of upstream regulatory molecules including transcription factors (TF) and master regulators (MRs), which are positioned at the top of the regulatory hierarchy [38]. As such, modulation of MR activity through drug treatment poses as an attractive therapeutic avenue in targeting multiple dysregulated downstream pathways in cancer [39]. To this purpose, we carried out *in silico* analysis using the *GeneXplain* platform to identify regulators responsible for the observed differential expression patterns of belinostat and Cubisbel [40].

Firstly, 110 different TFs were identified whose binding sites (motifs) were over-represented in promoters of DEGs dysregulated by HDAC inhibition in all colon cancer cell lines (Supplementary Table 5). Namely, RELA, MYOG, LEF-1 and TGIF were among TFs commonly found in promoters of genes dysregulated by belinostat and Cubisbel in the SW480-SW620 paired cell line, while highest enrichment for the JUN-D binding sites was identified in genes dysregulated by both drugs in CACO-2 cells.

**Fig. 4:**
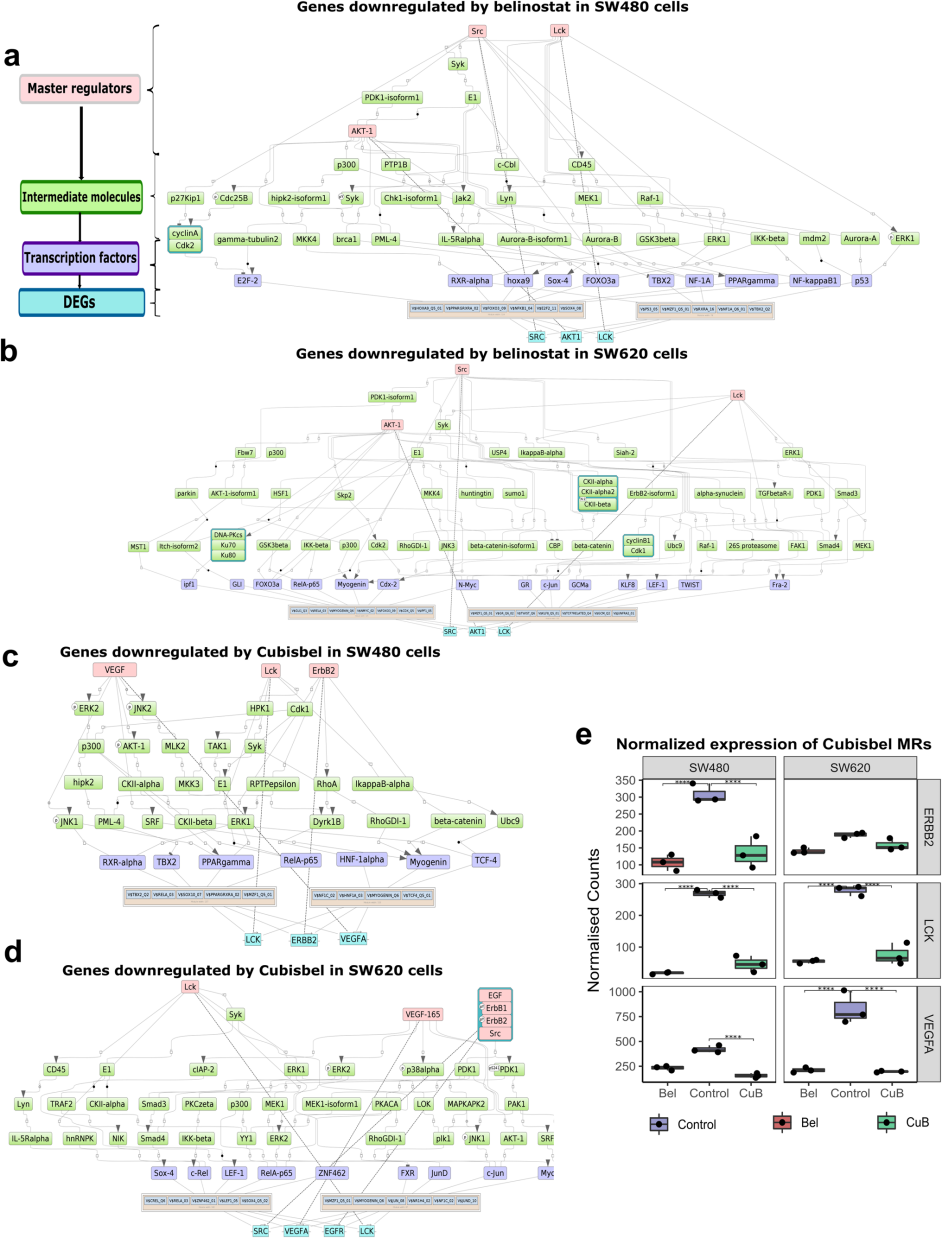
Identification of intracellular regulatory signalling pathways and MRs of genes dysregulated by belinostat and Cubisbel in SW480 and SW620 cells. **a**,** b** Intracellular signalling networks of DEGs downregulated by belinostat in SW480 and SW620 cells respectively. **c**,** d** DEGs downregulated by Cubisbel in SW480 and SW620 cells respectively, identified through *in silico* analysis of DEGs. Master regulatory molecules are indicated by pink rectangles, intermediate molecules are green and transcription factors are indicated by purple. Enriched binding motifs are highlighted by a green/orange border while example genes from the input list are shown in blue. **e** Boxplots showing normalized expression counts of example top ranking MRs in SW480 and SW620 cells treated with belinostat and Cubisbel. Counts represent the mean of n = 3 biological replicates

Based on the identified TFBS/TFs, further upstream analysis revealed *AKT1* as the top common MR that regulates TFs enriched within genes downregulated by belinostat in SW480 and SW620 cells (total rank of 51 and 28 respectively, as described in the Methods) (Fig. 4a and b), while *PTPRH* commonly regulated TFs enriched within genes upregulated by belinostat and Cubisbel (Supplementary Fig. 11b-d). In both cell lines, *LCK* and *ERBB2* consistently appeared as top ranking MRs of genes downregulated by Cubisbel in comparison to belinostat (Fig. 4c and d). Uniquely, *VEGFA* (specifically VEGF-165*)* was the only MR of genes downregulated by Cubisbel and not belinostat (Supplementary Table 6), *via* intermediary molecules such as *ERK2*. Subsequent reference to gene expression data confirmed significant downregulation of each of the above MRs in SW480 and SW620 cells by Cubisbel and additionally belinostat (Fig. 4e). This suggested that while the genes expressing these MRs are similarly altered by both HDACi, those such as *VEGFA* only act as MRs *via* control of TFs enriched within genes dysregulated specifically by Cubisbel.

**Fig. 5:**
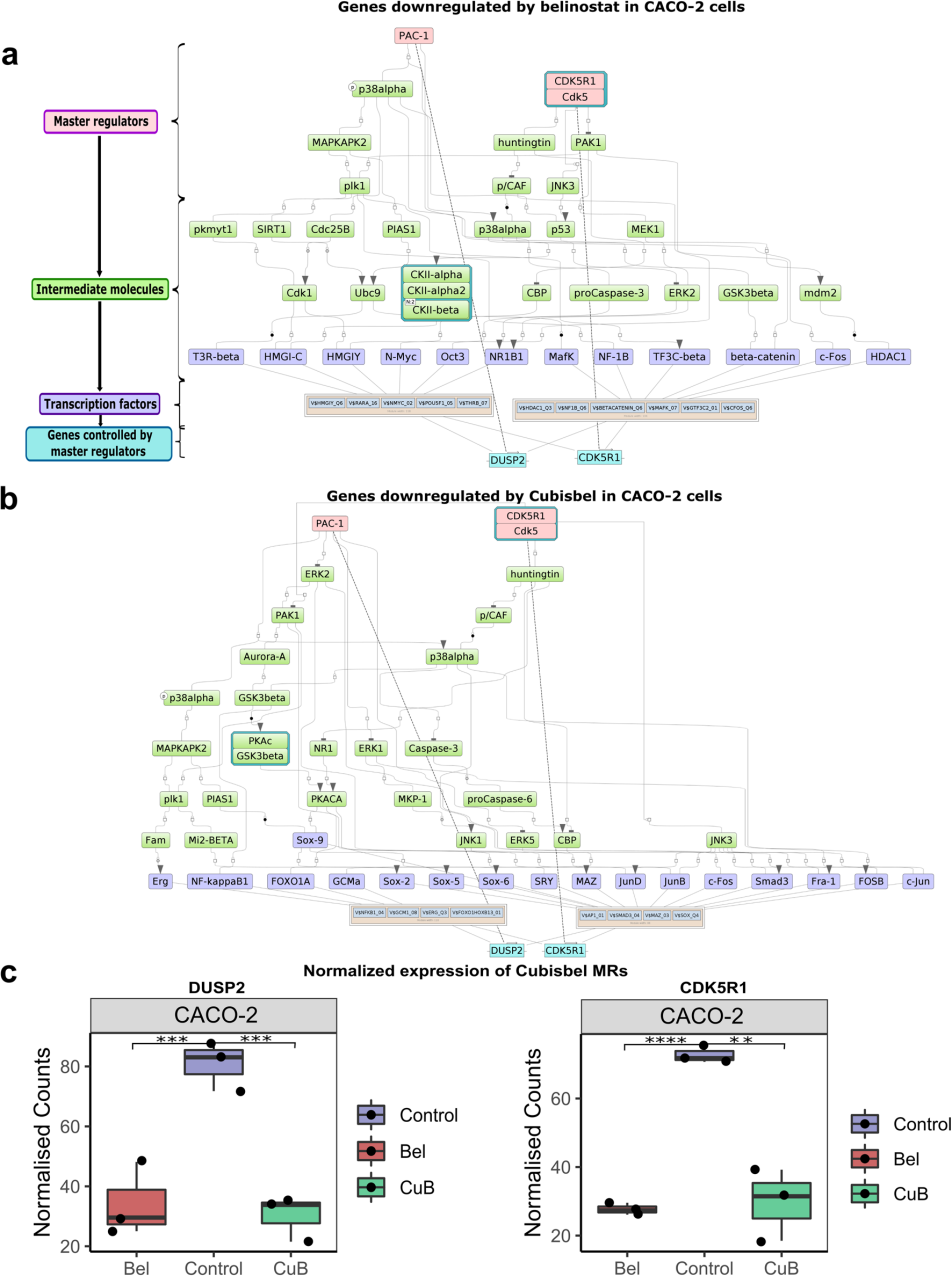
Identification of intracellular regulatory signalling pathways and MRs of genes dysregulated by belinostat and Cubisbel in CACO-2 cells. **a**,** b** Intracellular signalling networks of DEGs downregulated by belinostat and Cubisbel respectively in CACO-2 cells, identified through *in silico* analysis of DEGs. Master regulatory molecules are indicated by pink rectangles, intermediate molecules are green and transcription factors are indicated by purple. Enriched binding motifs are highlighted by a green/orange border while example genes from the input list are shown in blue. **c** Boxplots showing normalized expression counts of example MRs in CACO-2 cells treated with belinostat and Cubisbel. Counts represent the mean of n = 3 biological replicates

Additionally, in CACO-2 cells, *CDK5/CDK5R1* was identified as a key MR for genes commonly downregulated by belinostat and Cubisbel (Fig. 5), while *FYN* was uniquely involved in the signalling network for genes upregulated by belinostat (Supplementary Fig. 11e). Uniquely, PAC-1 (*DUSP2*) was the top ranking MR of genes downregulated by Cubisbel in this cell line. No MRs for genes significantly upregulated by Cubisbel were found in CACO-2 cells.

In summary, we identified specific signalling pathways regulated by MRs, which in turn mediate DEGs through specific TFs in response to belinostat or Cubisbel across all three colon cancer cell lines. Importantly, we show that when compared to belinostat, Cubisbel DEGs are potentially regulated by additional colon cancer-associated MRs [37].

### Belinostat and copper-bis-belinostat are potent inhibitors of proliferation and stemness in colon cancer patient-derived tumor organoids

**Fig. 6:**
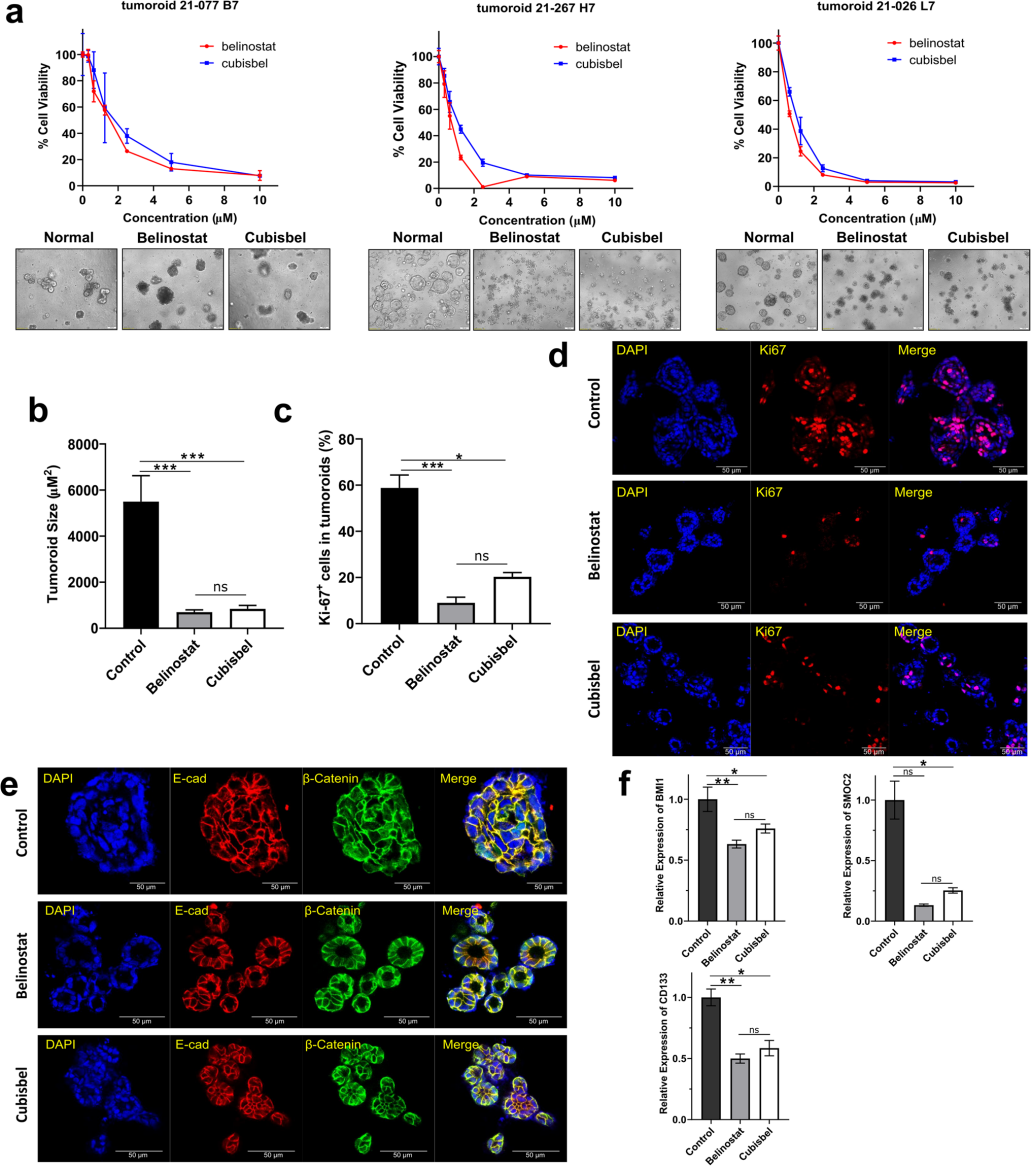
Examination of belinostat an Cubisbel activity in three colon cancer PDTOs. **a** Effect of belinostat and Cubisbel on the viability of three colon cancer PDTOs determined using the CellTiter-Glo 3D assay. Representative brightfield images of untreated PDTOs and PDTOs treated with 2.5 µM of HDACis are shown. **b** PDTO 21–267 H7 size (µm^2^) following treatment with either HDACi. **c** Quantification of Ki67 percentage staining in PDTO 21–267 H7 treatment analyzed using ImageJ software. **d** Corresponding representative 2D images of HDACi-treated PDTO 21–267 H7. Blue: DAPI, Red: Ki67. **e** Immunofluorescence of E-Cadherin and β–catenin in the 3D culture of PDTO 21–267 H7 treated with each HDACi. **f** Gene expression analysis of stem cell markers in PDTO 21–267 H7 following HDACi treatment detected using RT-qPCR. Results are presented as relative mRNA expression levels using the 2-ΔΔCT method after normalizing to GAPDH. For all experiments PDTOs were treated with 72 h IC_50_ concentrations of each HDACi. Scale bar = 50 µm

Cancer organoids are an emerging platform for informing clinical decisions and assessing drug sensitivity [41]. As such, to validate the effects of belinostat and Cubisbel on colon cancer cell viability in an independent pre-clinical model system, we next treated three colon cancer patient-derived tumor organoids (PDTOs) with varying clinical features (Supplementary Table 2) with the HDACis. After 72 h, treatment with each HDACi resulted in a large reduction in colon cancer organoid viability (Fig. 6a), with resulting IC_50_ values in the low micromolar range for both drugs, comparable to those in the colon cancer cell lines (Table 3). Surprisingly, the IC_50_ of Cubisbel was significantly higher than belinostat (*P* = 0.035) in the 21–267-H7 PDTO, indicating lower potency. However, no difference in IC_50_ concentrations was observed between the HDACis across the two additional PDTOs. In parallel, distinct morphological changes in the organoids were observed at all concentrations greater than the IC_50_ (Supplementary Fig. 12). Measurement of one colon cancer PDTO (21–267 H7) after IC_50_ treatment showed a significant size decrease, similar across treatments with belinostat (*P* = 0.00045) and Cubisbel (*P* = 0.00056) (Fig. 6b). Subsequent confocal imaging of the colon cancer PDTO confirmed the anti-proliferative activity of belinostat and Cubisbel marked by the reduction in Ki67 expression following treatment (*P* = 0.0008 and 0.013 respectively) (Fig. 6c and d).

**Table 3:** IC_50_ concentrations of belinostat and Cubisbel in colon cancer PDTOs

	Mean IC_50_ (µM ± SD)	
Colon cancer PDTO	Belinostat	Cubisbel
*21–267-H7*	0.667 ± 0.16	1.030 ± 0.21*
*21–077-B7*	1.413 ± 0.04	1.808 ± 0.69
*21–026-L7*	0.636 ± 0.05	1.925 ± 0.16

Using the 72 h time-point of colon cancer PDTOs treated with belinostat or Cubisbel, the IC_50_ was calculated using Prism (Graphpad v8.3.1). Absorbency values were averaged and transformed to the logarithmic values. Hence the non-linear fit function was applied to produce IC_50_ values (log(inhibitor) vs. normalized response—variable slope). Statistical significance was calculated using the student’s t-test (*P* ≤ 0.05), with each data point representing the mean IC_50_ obtained from triplicate wells ± SD. Values marked with an asterisk ‘*’ (*P* ≤ 0.05) indicate a significant difference between belinostat and Cubisbel IC_50_ concentrations

It has previously been shown that HDAC enzymes play a critical roles in the cancer stem cell (CSC) phenotype and epithelial-mesenchymal transition (EMT) [42, 43]. As PDTOs recapitulate, to a great extent, the true *in vivo* tumor environment compared to cell lines, we investigated the impact of the HDACi on these phenotypes in the PDTOs as an additional point of interest. Immunostaining for EMT markers E-cadherin and β-catenin was first carried out as shown in the 3D images in Fig. 6e. A stronger intensity for β–catenin staining alone was observed in organoids treated with DMSO control versus belinostat or Cubisbel (Supplementary Fig. 13). We demonstrated that when compared to the vehicle control, treatment of the 21–267 H7 organoid with belinostat and Cubisbel resulted in downregulation of a panel of stem cell markers including *BMI1, CD133* and *SMOC2* (*BMI1* *P* = 0.004 and 0.017; *CD133* *P* = 0.001 and 0.013; *SMOC2* *P* = ns and *P* = 0.011 respectively) (Fig. 6f). This is in line with the effects of the HDACis on these genes in SW480 and SW620 cells (Supplementary Fig. 14). Taken together, the potent effects of both the HDACi belinostat and its Cu(II) derivative on patient-derived colon cancer PDTO proliferation, morphology and pro-tumorigenic markers rationalize the enormous potential of the novel Cu-based prodrug in the treatment of colon cancer.

## Discussion

Despite the promising utility of HDAC inhibitors in the treatment of haematologic malignancies, therapeutic outcomes of HDACis in solid tumors, such as colon cancer, have so far been limited. This is likely owed to the susceptibility of the hydroxamic acid group of HDACis, such as belinostat, to rapid phase II metabolism, meaning that many HDACis lack sufficient stability to reach solid tumor cells [13, 15].

Here, we demonstrated that development of a novel Cu-based prodrug, Cubisbel, generated through complexation of clinically used anti-neoplastic agent belinostat to Cu(II), *via* binding directly to the hydroxamate moiety of belinostat, provides protection against metabolic inactivation *in vitro.* For the first time, we report both the phenotypic and molecular alterations induced by belinostat and novel prodrug Cubisbel in both colon cancer cells and patient-derived tumor organoids (PDTOs) and showed that complexation with Cu(II) did not prevent the mechanism of action of belinostat, but instead provided potent inhibition of cell growth and HDAC activity. Furthermore, data presented in this study provides a comprehensive understanding of the molecular alterations induced by these HDACis in colon cancer and therefore provide strong evidence for evaluation of the pharmacokinetic effects of Cubisbel *in vivo* as well as potential drug combinations for this HDACi for the treatment of colon cancer.

One remarkable finding from our study was that Cubisbel showed far superior predicted *in vitro* metabolic stability to belinostat in human liver microsomes (HLMs) and a significantly reduced predicted intrinsic clearance rate. As it has been previously been shown that at low concentrations, Cu(II) does not impact UGT enzyme activity, primarily responsible for belinostat metabolism, this finding confirmed our rationale that complexation to Cu(II) can indeed protect the hydroxamate moiety of belinostat from metabolic degradation [44]. As HLMs have proven successful in predicting *in vivo* pharmacokinetics, our *in vitro* data provides a basis for future prediction of intrinsic hepatic clearance *in vivo* [22, 45].

Similar to the findings of previous *in vitro* studies involving application of belinostat in hepatocellular carcinoma (HCC) [7], bladder cancer [46] and prostate cancer [8], both belinostat and novel Cubisbel significantly inhibited the growth of colon cancer cells in the low micromolar range after 72 h (IC_50_ 0.26 – 2 μM). As the three colon cancer cell lines possess underlying p53-mutations, this is in line with previously demonstrated selectivity of Cu(II) complexes incorporating HDACis to p53-mutant cell lines [17]. Remarkably, Cubisbel was significantly more potent in SW480 cells compared to belinostat, with an IC_50_ half that of belinostat. Previously, we have shown that Cu(II)-containing HDAC prodrugs can mediate DNA damage by producing reactive oxygen species, which may be one possible explanation for the greater impact on cell growth by Cubisbel in this cell line [47]. While it remains unclear whether the increased potency of Cubisbel was due to synergy provided by the Cu(II) ion or the increased dosage of belinostat provided by Cubisbel, it is possible that underlying molecular traits of the SW480 cell line influenced the increased sensitivity to Cubisbel.

The cytotoxic effect of belinostat and Cubisbel can likely be explained by the strong HDAC inhibition and apoptosis induction observed across all three colon cancer cell lines [13]. The effective reduction in overall HDAC activity by Cubisbel also confirmed successful separation of active drug belinostat from the Cu(II) ion in the reducing environment of colon cancer cells for the first time, rendering belinostat free to exhibit its HDAC inhibitory effect. This is in line with findings from previous studies in pancreatic cancer and acute myeloid leukemia (AML) [48, 49]. Furthermore, we found that Cu(II) present in the Cubisbel prodrug does not impact HDAC activity, which is in line with previous literature [50].

As histone modifications play a key role in the regulation of gene expression and cell cycle progression, it was surprising that cell cycle alterations were not consistent across all three colon cancer cell lines [51]. The G2-phase accumulation observed in CACO-2 cells, however, coupled with the induction of lower levels of apoptosis when compared to SW480 and SW620 cells, thus suggests a more cytostatic effect of Cubisbel in the CACO-2 cell line [52]. It is likely that the divergent pattern of response between the colon cancer cell lines in this instance was dependent on the integrity of their underlying cell cycle checkpoints [53, 54]. Ultimately, the subtle phenotypic differences observed between belinostat and Cubisbel treatments in this study were evident in a cell line-specific manner and were likely owed to heterogeneous intrinsic transcriptional/molecular processes that impact sensitivity to the HDACis.

Until now, the effect of belinostat on the colon cancer transcriptome remained unknown. By carrying out RNA-Seq analysis, we primarily found a large overlap of commonly dysregulated genes between belinostat and Cubisbel in all colon cancer cell lines, highlighting that the intrinsic molecular activity of belinostat was maintained, despite complexation to Cu(II). Furthermore, numerous metabolic processes were consistently downregulated by both HDACis in all colon cancer cells, in line with the emerging role of HDACis in cancer cell metabolic reprogramming [55].

Upon examination of the unique molecular alterations induced by Cubisbel alone, we identified *CD22* and *ZNF114* as genes uniquely downregulated by Cubisbel in SW620 cells. It has been reported that *CD22* functions as a regulator of B-cell response and is associated with oxaliplatin resistance in colon cancer, while *ZNF114* potentially promotes non-small cell lung cancer (NSCLC) metastasis [56–58]. Furthermore, Cubisbel uniquely upregulated genes such as *NTRK2* in SW480 cells, which has been previously suggested to act as a tumor suppressor in colorectal cancer [59]. Unique regulation of such cancer-associated genes by Cubisbel thus highlights its potential favorable utility in the treatment of colon cancer.

It is reported that key regulatory factors controlled by therapeutic agents may ultimately mediate altered gene expression [60]. To that end, our *in silico* analysis revealed that downregulation of genes by belinostat and Cubisbel is commonly mediated *via* TFs such as RELA and MYO-G in SW480 and SW620 cells. Interestingly, these appeared to signal through divergent upstream regulatory networks such as those controlled by MR *AKT1* for genes downregulated by belinostat and *VEGFA *and *ERBB2 *for genes downregulated by Cubisbel. The AKT1/PI3K pathway is activated in colon cancer by various mechanisms, whereas *ERBB* signaling has previously shown enrichment by belinostat in breast cancer cells [36, 61]. Evidence of *VEGFA* as the sole MR unique to Cubisbel potentially suggests a clinical advantage for the combination of Cubisbel with targeted anti-*VEGF* therapies routinely used to treat colon cancer such as bevacizumab [62]. In contrast to SW480 and SW620 cell lines, we observed that genes downregulated by Cubisbel in CACO-2 cells were controlled by *DUSP2,* which knowingly inactivates MAPK signaling by dephosphorylating *ERK* and *AKT *[63]*.*

Cumulatively, this *in silico* analysis for the first time identified the upstream regulatory pathways controlled not only by the novel prodrug, but also by belinostat in colon cancer. Commonly, the aforementioned MRs all have known involvement with different parts of the MAPK/ERK signaling cascade, which is activated in over 40% of colon cancer cases, thus rationalizing the use of targeted anti-EGFR / *VEGF* therapies in combination with such HDACis [62, 64].

Organoid cultures have many advantages over traditional 2D cell lines as they retain their phenotypic/genetic stability and heterogeneity of their original tissues, closely mimicking cellular diversity *in vivo* [65]*.* To our knowledge, this is the first time the activity of belinostat, in addition to the novel Cu-based prodrug, has been explored in colon cancer PDTOs. Promisingly, IC_50_ concentrations of the HDACis were in the low micromolar range alike the colon cancer cell lines with little variation between these concentrations. Surprisingly, the IC_50_ of Cubisbel was significantly higher than belinostat in the 21–267-H7 PDTO. Potentially, release of active belinostat from the Cubisbel prodrug may have only been partial in this *ex vivo* system or the PDTO was less sensitive due to underlying inter-patient heterogeneity, similar to what was observed in the colon cancer cells.

While the consistent pattern of response of the PDTOs to Cubisbel is highly encouraging, future work should include RNA-Seq of colon cancer PDTOs treated with these HDACis to better understand variation at the transcriptomic level to better predict interpatient drug sensitivities and guide precision medicine [66]. Finally, intestinal stem cell markers are often overexpressed in malignancies such as colon cancer and are associated with metastasis and chemoresistance [67]. Here, as an additional point of interest, we showed that both Cubisbel and belinostat can downregulate expression of *BMI1*, *SMOC2* and *CD133* in one PDTO, consistent with the effects of belinostat and Cubisbel on SW480 and SW620 cells. This further substantiates the broad anti-cancer mechanism of these HDACis in colon cancer.

## Conclusion

**Fig. 7:**
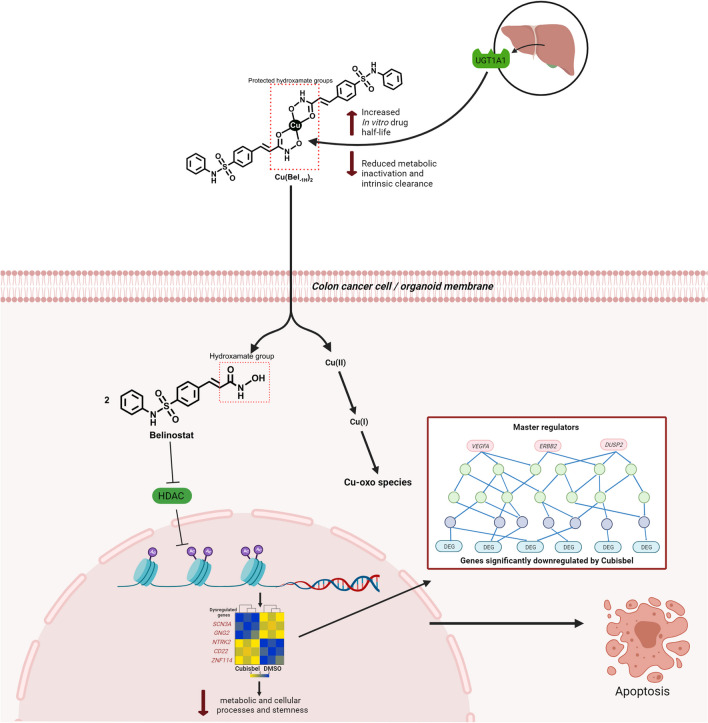
Schematic illustration of the potential mechanism of Cubisbel in colon cancer. In brief, our study has identified that complexation of a Cu(II) ion to belinostat *via* its hydroxamate moiety significantly slows the rapid metabolic degradation of belinostat *in vitro* by the UGT1A1 liver enzyme. We propose that upon entry to the reducing cellular environment, Cu(II) is reduced to Cu(I), allowing the resulting free belinostat to undergo its potent HDAC inhibitory and cytotoxic effect. This occurs *via* the upregulation of apoptosis and downregulation of multiple pathways and genes controlled by MRs involved in MAPK/ERK signaling

In conclusion, our study for the first time presents significant evidence to support complexation of belinostat to Cu(II) with the aim of improving the half-life of this HDACi in tumor cells, ultimately enhancing its anti-cancer potential and thus making it a plausible therapeutic option for colon cancer by preventing early metabolic degradation. Importantly, our data provides a detailed insight into the molecular regulatory landscape encompassing MR-TF controlled gene expression changes, underpinning the anti-cancer phenotype mediated by Cubisbel and belinostat (Fig. 7). This anti-cancer phenotype of Cubisbel was validated in colon cancer PDTOs where potent inhibition of cell proliferation and stemness was also observed.

While we acknowledge that the lack of *in vivo* work is the primary limitation of this study, the *in vitro* and *ex vivo* derived results outlined in this study provide clear rationale for further evaluation of Cubisbel through extensive *in vivo* studies using orthotopic PDX-based colon cancer models. These will be pivotal in confirming the pharmacokinetics and toxicity of Cubisbel and its potential as a monotherapy or as a combination therapy with the aforementioned standard of care in colon cancer. As such, phenotypes including tumor cell growth, death and stemness impacted by the HDACi treatment in this study also warrant further examination *in vivo*.

Although Cubisbel showed subtle phenotypic differences in comparison to clinically approved parent drug belinostat, it is possible that this would be different in *in vivo* models given that belinostat routinely undergoes rapid metabolic degradation by the UGT1A1 liver enzyme not present in *in vitro / ex vivo* cell line models, which we acknowledge as a limitation of this study. Nevertheless, we have demonstrated the possibility of prolonging HDACi half-life *in vitro* through complexation with a metal ion while maintaining the cellular and molecular impact of belinostat. Based on this and our previous work on metallo-prodrugs in cancer, we propose a novel strategy with significant potential to revisit the use of HDACis as improved therapeutics for the treatment of solid tumors, which has so far had very limited success [17].

### Supplementary Information

Below is the link to the electronic supplementary material.

Supplementary file1 (DOCX 25234 KB)

Supplementary file2 (DOCX 26 KB)

Supplementary file3 (DOCX 22 KB)

Supplementary file4 (XLSX 3558 KB)

Supplementary file5 (XLSX 220 KB)

Supplementary file6 (XLSX 51 KB)

Supplementary file7 (XLSX 50 KB)

## Data Availability

The data generated and/or analyzed for this study are included in the published article and supplementary information files.

## Abbreviations

Cubisbel: Copper-bis-belinostat
DEG: Differentially expressed gene
HDACi: Histone deacetylase inhibitor
HLM: Human liver microsome
MR: Master regulator
PDTO: Patient-derived tumor organoid
SAHA: Suberoylanilide hydroxamic acid
TFBS: Transcription factor binding site

## References

[1] Rawla P, Sunkara T, Barsouk A (2019). Epidemiology of colorectal cancer: incidence, mortality, survival, and risk factors. Prz Gastroenterol..

[2] Wang J, Li S, Liu Y, Zhang C, Li H, Lai B (2020). Metastatic patterns and survival outcomes in patients with stage IV colon cancer: A population-based analysis. Cancer Med..

[3] Y. Li, E. Seto, HDACs and HDAC Inhibitors in Cancer Development and Therapy. Cold Spring Harb Perspect Med. **6**(10), a026831 (2016) 10.1101/cshperspect.a026831 PMC5046688 27599530

[4] Yang H, Salz T, Zajac-Kaye M, Liao D, Huang S, Qiu Y (2014). Overexpression of histone deacetylases in cancer cells is controlled by interplay of transcription factors and epigenetic modulators. FASEB J. Off. Publ. Fed. Am. Soc. Exp. Biol..

[5] Deubzer HE, Schier MC, Oehme I, Lodrini M, Haendler B, Sommer A (2013). HDAC11 is a novel drug target in carcinomas. Int. J. Cancer.

[6] H.-Z. Lee, V.E. Kwitkowski, P.L. Del Valle, M.S. Ricci, H. Saber, B.A. Habtemariam, J. Bullock, E. Bloomquist, Y. Li Shen , X.H. Chen, J. Brown, N. Mehrotra, S. Dorff, R. Charlab, R.C. Kane, E. Kaminskas, R. Justice, A.T. Farrell, R. Pazdur. FDA Approval: Belinostat for the Treatment of Patients with Relapsed or Refractory Peripheral T-cell Lymphoma. Clin Cancer Res. **21**(12), 2666–2670 (2015) 10.1158/1078-0432.CCR-14-3119 25802282

[7] Young MD, Wakefield MJ, Smyth GK, Oshlack A (2010). Gene ontology analysis for RNA-seq: accounting for selection bias. Genome Biol..

[8] Qian X, Ara G, Mills E, LaRochelle WJ, Lichenstein HS, Jeffers M (2008). Activity of the histone deacetylase inhibitor belinostat (PXD101) in preclinical models of prostate cancer. Int. J. Cancer.

[9] Juergens RA, Wrangle J, Vendetti FP, Murphy SC, Zhao M, Coleman B (2011). Combination epigenetic therapy has efficacy in patients with refractory advanced non-small cell lung cancer. Cancer Discov..

[10] Lassen U, Molife LR, Sorensen M, Engelholm SA, Vidal L, Sinha R (2010). A phase I study of the safety and pharmacokinetics of the histone deacetylase inhibitor belinostat administered in combination with carboplatin and/or paclitaxel in patients with solid tumours. Br. J. Cancer.

[11] W. K. Kelly, J. DeBono, G. Blumenschein, U. Lassen, J. Zain, O. O'Connor, F. Foss, J. Tjornelund, J. Fagerberg, and D. Petrylak. Final results of a phase I study of oral belinostat (PXD101) in patients with solid tumors. J Clin Oncol **27**(15_suppl), 3531 (2009)

[12] Mackay HJ, Hirte H, Colgan T, Covens A, MacAlpine K, Grenci P (2010). Phase II trial of the histone deacetylase inhibitor belinostat in women with platinum resistant epithelial ovarian cancer and micropapillary (LMP) ovarian tumours. Eur. J, Cancer (Oxford, England : 1990).

[13] Mottamal M, Zheng S, Huang TL, Wang G (2015). Histone deacetylase inhibitors in clinical studies as templates for new anticancer agents. Molecules (Basel, Switzerland)..

[14] M.H. Cheng, J.Y. Weng, C.H. Chuang, W.T. Liao, Y.F. Lai, J.Y. Liu, et al., Prolonging the Half-Life of Histone Deacetylase Inhibitor Belinostat via 50 nm Scale Liposomal Subcutaneous Delivery System for Peripheral T-Cell Lymphoma. Cancers **12**(9) (2020) 10.3390/cancers12092558 PMC7563358 32911820

[15] Wang LZ, Ramírez J, Yeo W, Chan MY, Thuya WL, Lau JY (2013). Glucuronidation by UGT1A1 is the dominant pathway of the metabolic disposition of belinostat in liver cancer patients. PLoS ONE.

[16] Wang L-Z, Ramírez J, Yeo W, Chan M-YM, Thuya W-L, Lau J-YA (2013). Glucuronidation by UGT1A1 Is the Dominant Pathway of the Metabolic Disposition of Belinostat in Liver Cancer Patients. Plos One.

[17] McGivern TJP, Slator C, Kellett A, Marmion CJ (2018). Innovative DNA-Targeted Metallo-prodrug Strategy Combining Histone Deacetylase Inhibition with Oxidative Stress. Mol. Pharm..

[18] Griffith DM, Szocs B, Keogh T, Suponitsky KY, Farkas E, Buglyó P (2011). Suberoylanilide hydroxamic acid, a potent histone deacetylase inhibitor; its X-ray crystal structure and solid state and solution studies of its Zn(II), Ni(II), Cu(II) and Fe(III) complexes. J. Inorg. Biochem..

[19] C.J. Marmion, J.P. Parker, K.B. Nolan. Hydroxamic Acids: An Important Class of Metalloenzyme Inhibitors. In J. Reedijk, K. Poeppelmeier editors. Comprehensive Inorganic Chemistry II (Second Edition) **3**, 683–708 (2013)

[20] Daniel KB, Sullivan ED, Chen Y, Chan JC, Jennings PA, Fierke CA (2015). Dual-Mode HDAC Prodrug for Covalent Modification and Subsequent Inhibitor Release. J. Med. Chem..

[21] C. Zhang, S. Guo, Q. Zhong, Q. Zhang, A. Hossain, S. Zheng, Wang G. Metabolism and Pharmacokinetic Study of the Boron-Containing Prodrug of Belinostat (ZL277), a Pan HDAC Inhibitor with Enhanced Bioavailability. Pharmaceuticals (Basel, Switzerland) **12**(4), 180 (2019) 10.3390/ph12040180 PMC6958523 31817969

[22] Obach RS, Baxter JG, Liston TE, Silber BM, Jones BC, MacIntyre F (1997). The prediction of human pharmacokinetic parameters from preclinical and in vitro metabolism data. J. Pharmacol. Exp. Ther..

[23] Ewels PA, Peltzer A, Fillinger S, Patel H, Alneberg J, Wilm A (2020). The nf-core framework for community-curated bioinformatics pipelines. Nat. Biotechnol..

[24] S. A. FastQC: a quality control tool for high throughput sequence data (2010), Available from: http://www.bioinformatics.babraham.ac.uk/projects/fastqc. Accessed 15 Mar 2022

[25] F. K. Trimgalore (2021), Available from: GitHub repository, https://github.com/FelixKrueger/TrimGalore. Accessed 15 Mar 2022

[26] Dobin A, Davis CA, Schlesinger F, Drenkow J, Zaleski C, Jha S (2013). STAR: ultrafast universal RNA-seq aligner. Bioinformatics (Oxford, England)..

[27] Patro R, Duggal G, Love MI, Irizarry RA, Kingsford C (2017). Salmon provides fast and bias-aware quantification of transcript expression. Nat. Methods.

[28] Ozawa T, Matsuyama T, Toiyama Y, Takahashi N, Ishikawa T, Uetake H (2017). CCAT1 and CCAT2 long noncoding RNAs, located within the 8q.24.21 'gene desert', serve as important prognostic biomarkers in colorectal cancer. Ann. Oncol. Off. J. Eur. Soc. Med. Oncol..

[29] Love MI, Huber W, Anders S (2014). Moderated estimation of fold change and dispersion for RNA-seq data with DESeq2. Genome Biol..

[30] H W. ggplot2: Elegant Graphics for Data Analysis. Springer-Verlag New York (2016), Available from: https://ggplot2.tidyverse.org. Accessed 15 Mar 2022

[31] Gu Z, Eils R, Schlesner M (2016). Complex heatmaps reveal patterns and correlations in multidimensional genomic data. Bioinf. (Oxford, England).

[32] Matys V, Kel-Margoulis OV, Fricke E, Liebich I, Land S, Barre-Dirrie A (2006). TRANSFAC and its module TRANSCompel: transcriptional gene regulation in eukaryotes. Nucleic Acids Res..

[33] Waleev T, Shtokalo D, Konovalova T, Voss N, Cheremushkin E, Stegmaier P (2006). Composite Module Analyst: identification of transcription factor binding site combinations using genetic algorithm. Nucleic Acids Res..

[34] P.A. Myer, H. Kim, A.M. Blümel, E. Finnegan, A. Kel, T.V. Thompson, et al., Master transcription regulators and transcription factors regulate immune-associated differences between patients of African and European ancestry with Colorectal Cancer. Gastro Hep Adv. **1**(3), 328–341 (2022) 10.1016/j.gastha.2022.01.004 PMC9151447 35711675

[35] Kel AE, Stegmaier P, Valeev T, Koschmann J, Poroikov V, Kel-Margoulis OV (2016). Multi-omics "upstream analysis" of regulatory genomic regions helps identifying targets against methotrexate resistance of colon cancer. EuPA Open Proteom..

[36] Zuo Y, Xu H, Chen Z, Xiong F, Zhang B, Chen K (2020). 17-AAG synergizes with Belinostat to exhibit a negative effect on the proliferation and invasion of MDA-MB-231 breast cancer cells. Oncol. Rep..

[37] Yang Q, Feng M, Ma X, Li H, Xie W (2017). Gene expression profile comparison between colorectal cancer and adjacent normal tissues. Oncol. Lett..

[38] Sikdar S, Datta S (2017). A novel statistical approach for identification of the master regulator transcription factor. BMC Bioinformatics.

[39] Cai W, Zhou W, Han Z, Lei J, Zhuang J, Zhu P (2020). Master regulator genes and their impact on major diseases. PeerJ..

[40] Kel AE, Gössling E, Reuter I, Cheremushkin E, Kel-Margoulis OV, Wingender E (2003). MATCH: A tool for searching transcription factor binding sites in DNA sequences. Nucleic Acids Res..

[41] J. Liu, X. Huang, L. Huang, J. Huang, D. Liang, L. Liao, Y. Deng, L. Zhang, B. Zhang and W. Tang. Organoid: Next-Generation Modeling of Cancer Research and Drug Development. Front Oncol. **11**, 826613 (2022) 10.3389/fonc.2021.826613 PMC8831330 35155215

[42] Witt AE, Lee CW, Lee TI, Azzam DJ, Wang B, Caslini C (2017). Identification of a cancer stem cell-specific function for the histone deacetylases, HDAC1 and HDAC7, in breast and ovarian cancer. Oncogene.

[43] R. Hai, L. He, G. Shu, G. Yin Characterization of Histone Deacetylase Mechanisms in Cancer Development. Front Oncol. **11**, 700947 (2021) 10.3389/fonc.2021.700947 PMC8360675 34395273

[44] Letelier ME, Lagos F, Faúndez M, Miranda D, Montoya M, Aracena-Parks P (2007). Copper modifies liver microsomal UDP-glucuronyltransferase activity through different and opposite mechanisms. Chem. Biol. Interact..

[45] Nakamori F, Naritomi Y, Furutani M, Takamura F, Miura H, Murai H (2011). Correlation of Intrinsic in vitro and in vivo Clearance for Drugs Metabolized by Hepatic UDP-glucuronosyltransferases in Rats. Drug Metab. Pharmacokinet..

[46] Buckley MT, Yoon J, Yee H, Chiriboga L, Liebes L, Ara G (2007). The histone deacetylase inhibitor belinostat (PXD101) suppresses bladder cancer cell growth in vitro and in vivo. J. Transl. Med..

[47] McGivern TJP, Slator C, Kellett A, Marmion CJ (2018). Innovative DNA-Targeted Metallo-prodrug Strategy Combining Histone Deacetylase Inhibition with Oxidative Stress. Mol. Pharm..

[48] Dovzhanskiy DI, Arnold SM, Hackert T, Oehme I, Witt O, Felix K (2012). Experimental in vivo and in vitro treatment with a new histone deacetylase inhibitor belinostat inhibits the growth of pancreatic cancer. BMC Cancer.

[49] Stapnes C, Ryningen A, Hatfield K, Øyan AM, Eide GE, Corbascio M (2007). Functional characteristics and gene expression profiles of primary acute myeloid leukaemia cells identify patient subgroups that differ in susceptibility to histone deacetylase inhibitors. Int. J. Oncol..

[50] Kang J, Lin C, Chen J, Liu Q (2004). Copper induces histone hypoacetylation through directly inhibiting histone acetyltransferase activity. Chem. Biol. Interact..

[51] Telles E, Seto E (2012). Modulation of cell cycle regulators by HDACs. Front. Biosci. (Schol. Ed.).

[52] Marampon F, Di Nisio V, Pietrantoni I, Petragnano F, Fasciani I, Scicchitano BM (2019). Pro-differentiating and radiosensitizing effects of inhibiting HDACs by PXD-101 (Belinostat) in in vitro and in vivo models of human rhabdomyosarcoma cell lines. Cancer Lett..

[53] Ahmed D, Eide PW, Eilertsen IA, Danielsen SA, Eknæs M, Hektoen M (2013). Epigenetic and genetic features of 24 colon cancer cell lines. Oncogenesis..

[54] Waldman T, Zhang Y, Dillehay L, Yu J, Kinzler K, Vogelstein B (1997). Cell-cycle arrest versus cell death in cancer therapy. Nat. Med..

[55] R.K. Alseksek, W.S. Ramadan, E. Saleh, R. El-Awady, The Role of HDACs in the Response of Cancer Cells to Cellular Stress and the Potential for Therapeutic Intervention. Int. J. Mol. Sci. **23**(15), 8141 (2022) 10.3390/ijms23158141 PMC9331760 35897717

[56] E.A. Clark, N.V. Giltiay, CD22: A Regulator of Innate and Adaptive B Cell Responses and Autoimmunity. Front Immunol.** 9**, 2235 (2018) 10.3389/fimmu.2018.02235 PMC6173129 30323814

[57] Lin Q, Luo L, Wang H (2021). A New Oxaliplatin Resistance-Related Gene Signature With Strong Predicting Ability in Colon Cancer Identified by Comprehensive Profiling. Front. Oncol..

[58] Umeyama H, Iwadate M, Taguchi Yh (2014). TINAGL1 and B3GALNT1 are potential therapy target genes to suppress metastasis in non-small cell lung cancer. BMC Genom..

[59] Chen Z, Huang Z, Luo Y, Zou Q, Bai L, Tang G (2021). Genome-wide analysis identifies critical DNA methylations within NTRKs genes in colorectal cancer. J. Transl. Med..

[60] M.A. De Bastiani, B. Pfaffenseller, F. Klamt, Master Regulators Connectivity Map: A Transcription Factors-Centered Approach to Drug Repositioning;9 (2018) 10.3389/fphar.2018.00697 PMC6043797 30034338

[61] Hechtman JF, Sadowska J, Huse JT, Borsu L, Yaeger R, Shia J (2015). AKT1 E17K in Colorectal Carcinoma Is Associated with BRAF V600E but Not MSI-H Status: A Clinicopathologic Comparison to PIK3CA Helical and Kinase Domain Mutants. Mol. Cancer Res. MCR..

[62] Khanal N, Upadhyay S, Silberstein PT (2015). Colorectal Carcinoma and Emerging Targeted Therapies. Fed. Pract..

[63] Dong W, Li N, Pei X, Wu X (2018). Differential expression of DUSP2 in left- and right-sided colon cancer is associated with poor prognosis in colorectal cancer. Oncol. Lett..

[64] Blaj C, Schmidt EM, Lamprecht S, Hermeking H, Jung A, Kirchner T (2017). Oncogenic Effects of High MAPK Activity in Colorectal Cancer Mark Progenitor Cells and Persist Irrespective of RAS Mutations. Can. Res..

[65] Munro MJ, Tan ST, Gray C (2023). Applications for Colon. Organoid Models Cancer Res..

[66] Bruun J, Kryeziu K, Eide PW, Moosavi SH, Eilertsen IA, Langerud J (2020). Patient-Derived Organoids from Multiple Colorectal Cancer Liver Metastases Reveal Moderate Intra-patient Pharmacotranscriptomic Heterogeneity. Clin. Cancer Res..

[67] Zhou Y, Xia L, Wang H, Oyang L, Su M, Liu Q (2018). Cancer stem cells in progression of colorectal cancer. Oncotarget.

