# Supplementary material for: Complexation of histone deacetylase inhibitor belinostat to Cu(II) prevents premature metabolic inactivation in vitro and demonstrates potent anti-cancer activity in vitro and ex vivo in colon cancer
Source: Cell Oncol (Dordr). 2023 Nov 7;47(2):533–53. doi: 10.1007/s13402-023-00882-x (PMC11090832; doi:10.1007/s13402-023-00882-x)
Supplement: Supplementary file 1 — Supplementary file1 (DOCX 25234 KB) [file 13402_2023_882_MOESM1_ESM.docx]

**Supplementary Figures and Methods**

Supplementary Figures

**Supplementary Figure 1. Stability of Cubisbel in solution.** The stability of Cubisbel in 100% DMSO was examined in triplicate using the SHIMADZU spectrophotometer at various timepoints ranging from 0 to 10 days from 400 to 800 nm as shown on the spectra graph.

**
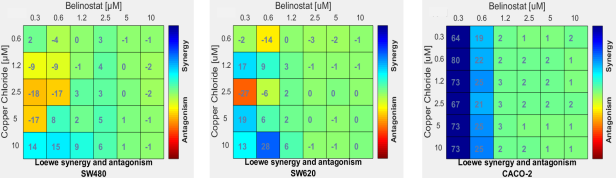
**

**Supplementary Figure 2. Synergy of belinostat and CuCl_2_ in colon cancer cells.** Synergy plots were generated using Combenefit Software showing the interaction between belinostat and CuCl_2_ in SW480, SW620 and CACO-2 cells 72 h after exposure to both drugs ranging from 0.3 µM to 10 µM. Data was analyzed for synergy and antagonism using the Loewe additivity mathematical model. Values > 0 indicate synergistic effects.

**
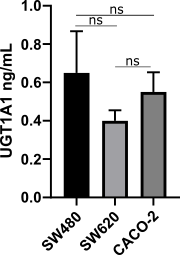
**

**Supplementary Figure 3. Baseline UGT1A1 protein expression in SW480, SW620 and CACO-2 cell lines.** Whole lysate protein extracts were prepared for all cell lines. UGT1A1 expression was measured by ELISA at 450nm. Each data point represents mean triplicate UGT1A1 expression (ng/ml) ± SD.


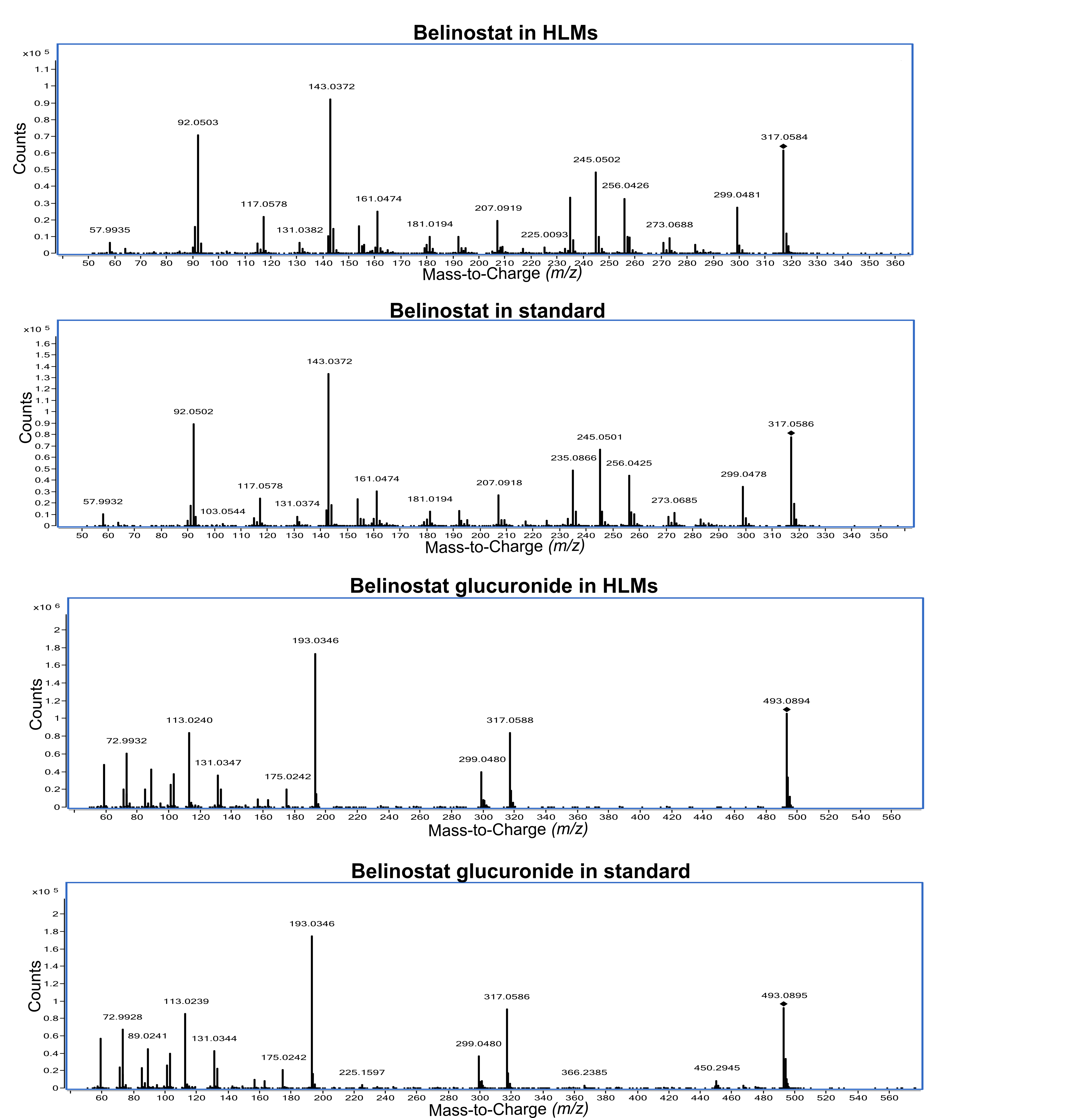


**Supplementary Figure 4. MS/MS spectra of belinostat and belinostat glucuronide in human liver microsome samples.** Representative MS/MS spectra plots of belinostat, belinostat glucuronide in HLM samples and corresponding standards at 20 collision energy.

**Supplementary Figure 5. Belinostat remaining in control microsomes without UGT cofactor addition.** The percentage of belinostat compound remaining in HLM samples was assessed after 0, 60 and 120 min incubation at 37 ^o^C with belinostat or Cubisbel HDACis.


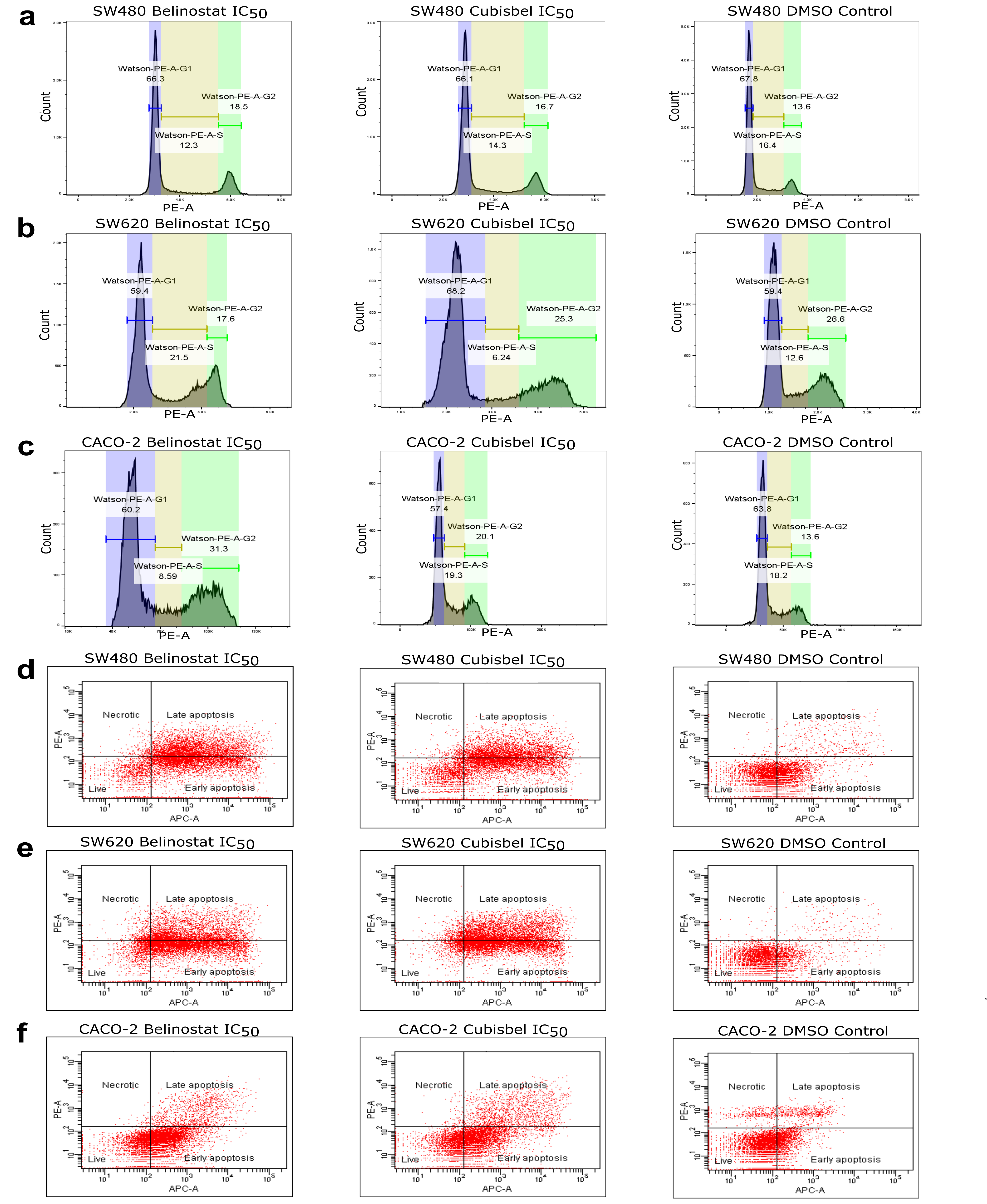


**Supplementary Figure 6. Flow Cytometry plots from cell cycle and apoptosis analysis of colon cancer cells treated with belinostat and Cubisbel.** Representative histograms of **(a)** SW480, **(b)** SW620 and **(c)** CACO-2 in different stages of the cell cycle generated using FlowJo software. Representative FACs scatter plots from annexin V/PI staining for apoptosis representing various cell populations for **(d)** SW480, **(e)** SW620 and **(f)** CACO-2 cells after HDACi treatment. Numbers represent percentage of cells present in each population.


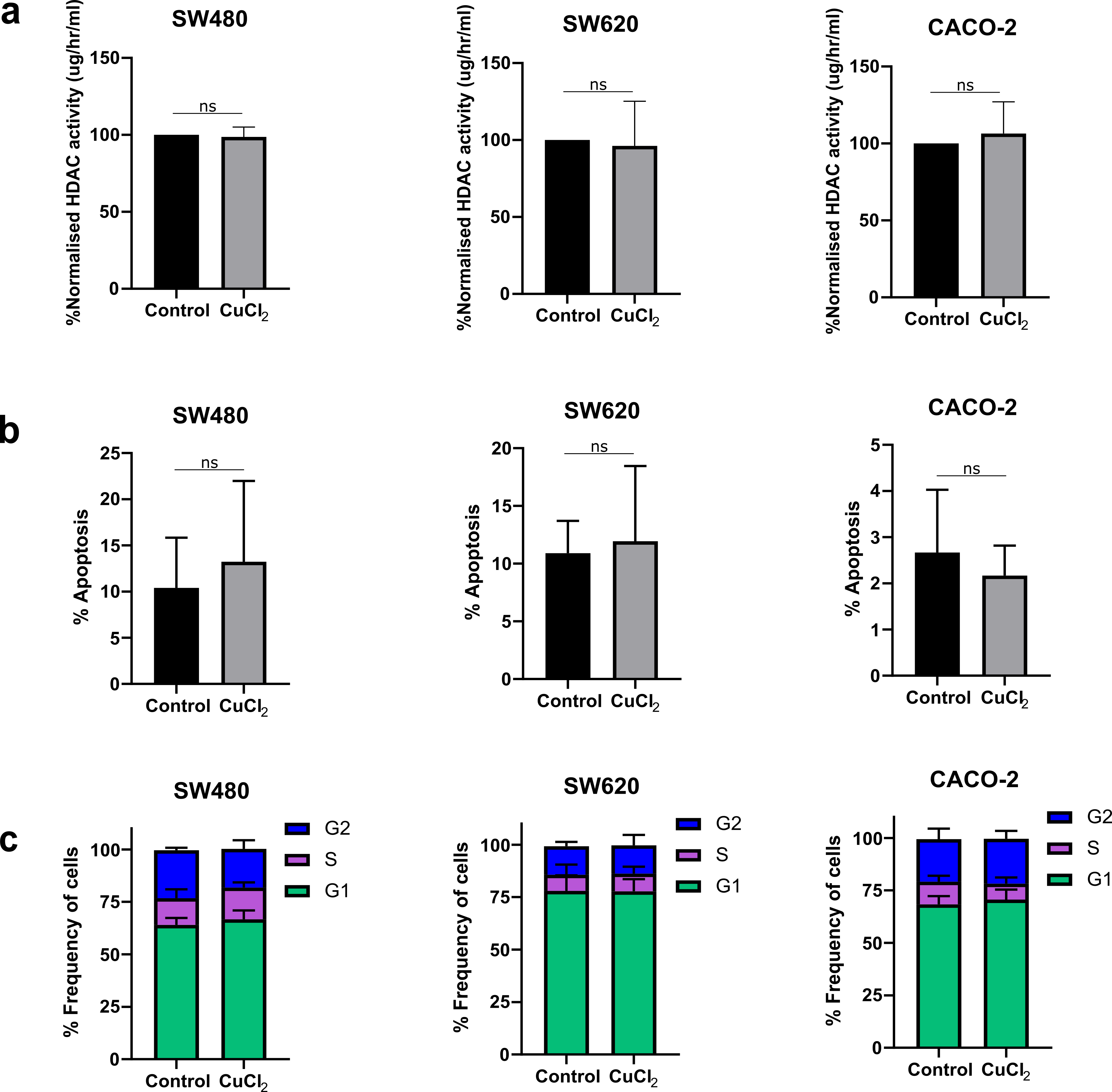


**Supplementary Figure 7. Effect of CuCl_2_ on colon cancer cell HDAC activity, apoptosis and cell cycle.** For all phenotypic assays, SW480, SW620 and CACO-2 cells were utilized 72 h post-treatment with CuCl_2_ at concentrations equivalent to that of Cubisbel IC_50_ (1.131, 1.412 and 0.369 µM respectively). **(a)** HDAC activity in colon cancer cells following CuCl_2_ treatment. Nuclear protein extracts were prepared using the EpiQuik nuclear extraction kit. HDAC activity was measured by the direct colorimetric assay kit EpiQuik (Epigentek). The resulting data was normalized to control cells treated with DMSO only, representing 100% HDAC activity (µg/h/ml). **(b)** Assessment of apoptosis using Annexin/PI staining following by flow cytometry. The percentage of early and late apoptotic cells (total apoptotic cells) compared to the negative control are illustrated by bar graphs for each cell line. **(c)** Percentage of cells in each cell cycle phase, graphically represented by stacked columns. The distribution of cells was analyzed by flow cytometry using PI to stain cell DNA. Cell populations in the G0/G1, S and G2/M phase are given as percentages of total cells. For all assays, statistical significance was calculated using the student’s t-test (p ≤ 0.05), with each data point representing the mean of n=3 biological replicates ± SD.


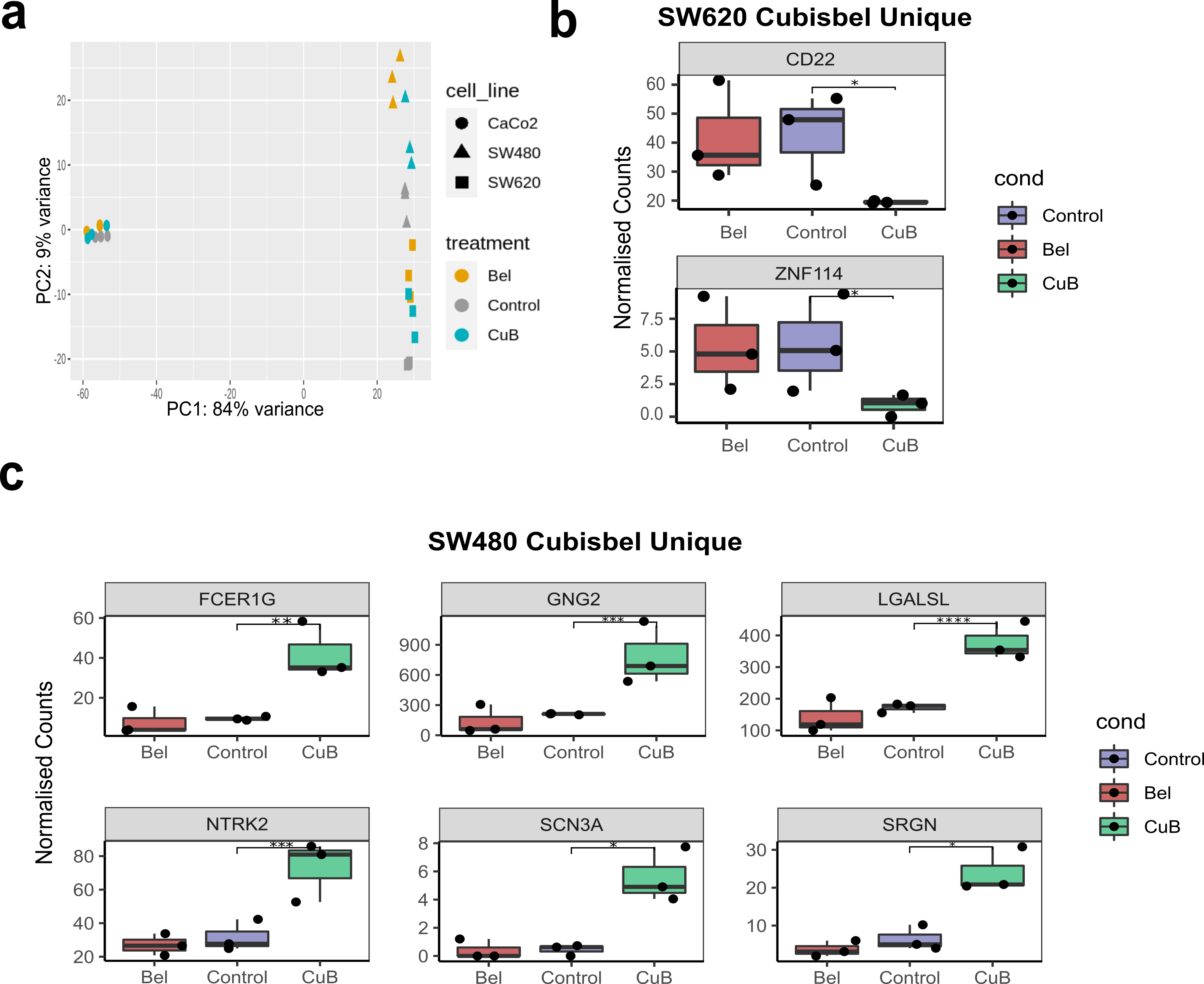


**Supplementary Figure 8. Analysis of genes dysregulated by belinostat and Cubisbel in colon cancer cell lines. (a)** Principal component analysis of RNA-Seq data for SW480, SW620 and CACO-2 cells treated with DMSO or IC_50_ concentrations of belinostat and Cubisbel for 72 h. Boxplots showing normalized expression counts of genes uniquely up- and downregulated by Cubisbel in **(b)** SW620 and **(c)** SW480 cells. Counts represent the mean of n=3 biological replicates. No unique DEGs were identified in CACO-2 cells treated with belinostat or Cubisbel.


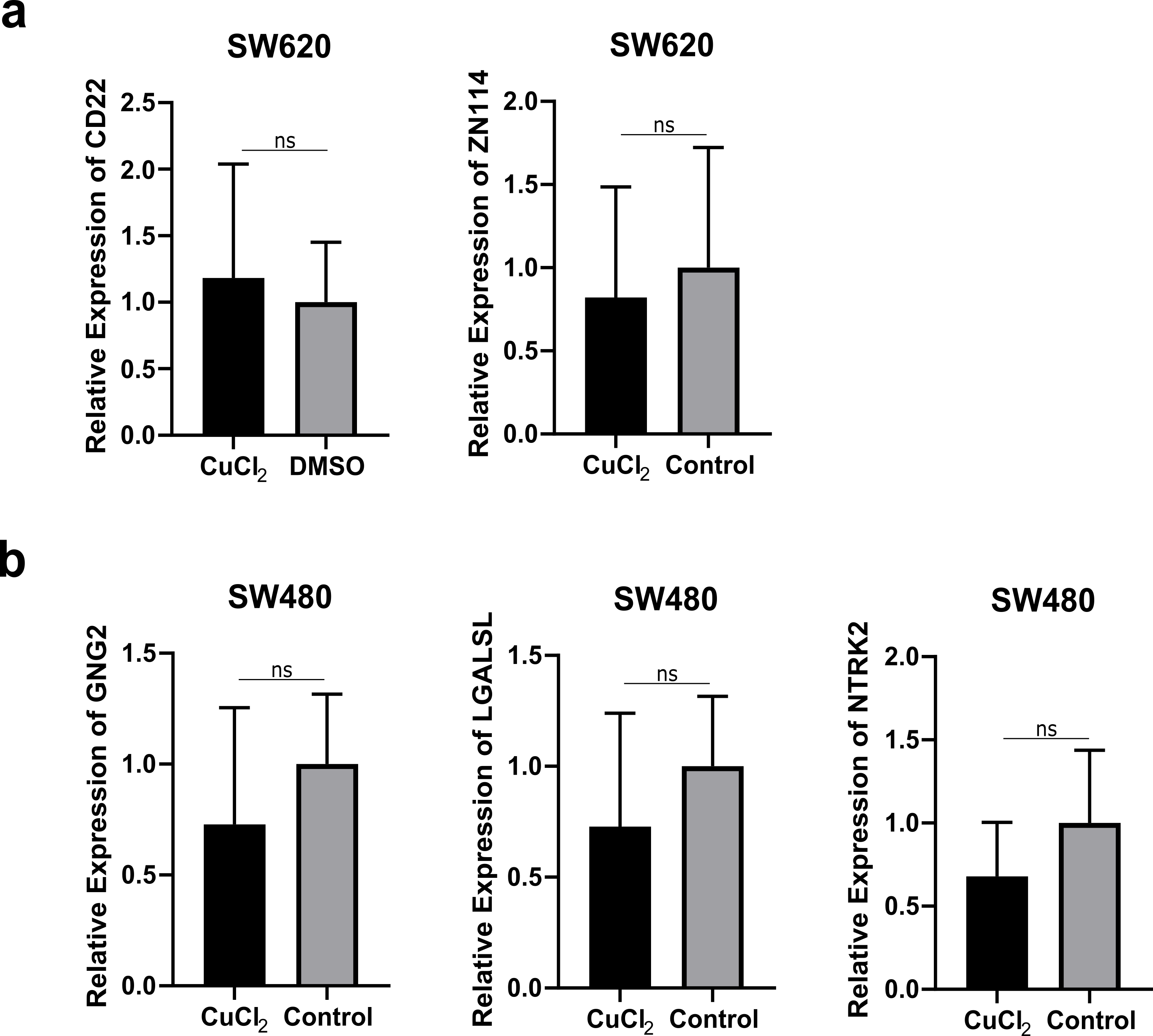


**Supplementary Figure 9. Impact of CuCl_2_ on genes uniquely regulated by Cubisbel in colon cancer cell lines.** mRNA levels of Cubisbel-unique genes **(a)** *CD22* and *ZNF114* identified in SW620 cells and top three genes **(b)** *GNG2*, *LGALSL* and *NTRK2* in SW480 cells following treatment with DMSO control or CuCl_2_ at equivalent Cubisbel 72 h IC_50_ concentrations. Gene expression was detected using RT-qPCR. Results were normalized to 18S expression and samples are shown relative to the DMSO control, which was also used in previous RNA-Seq analysis. Error bars represent SD of three independent experiments (n=3). Significance was calculated using a student’s t-test.


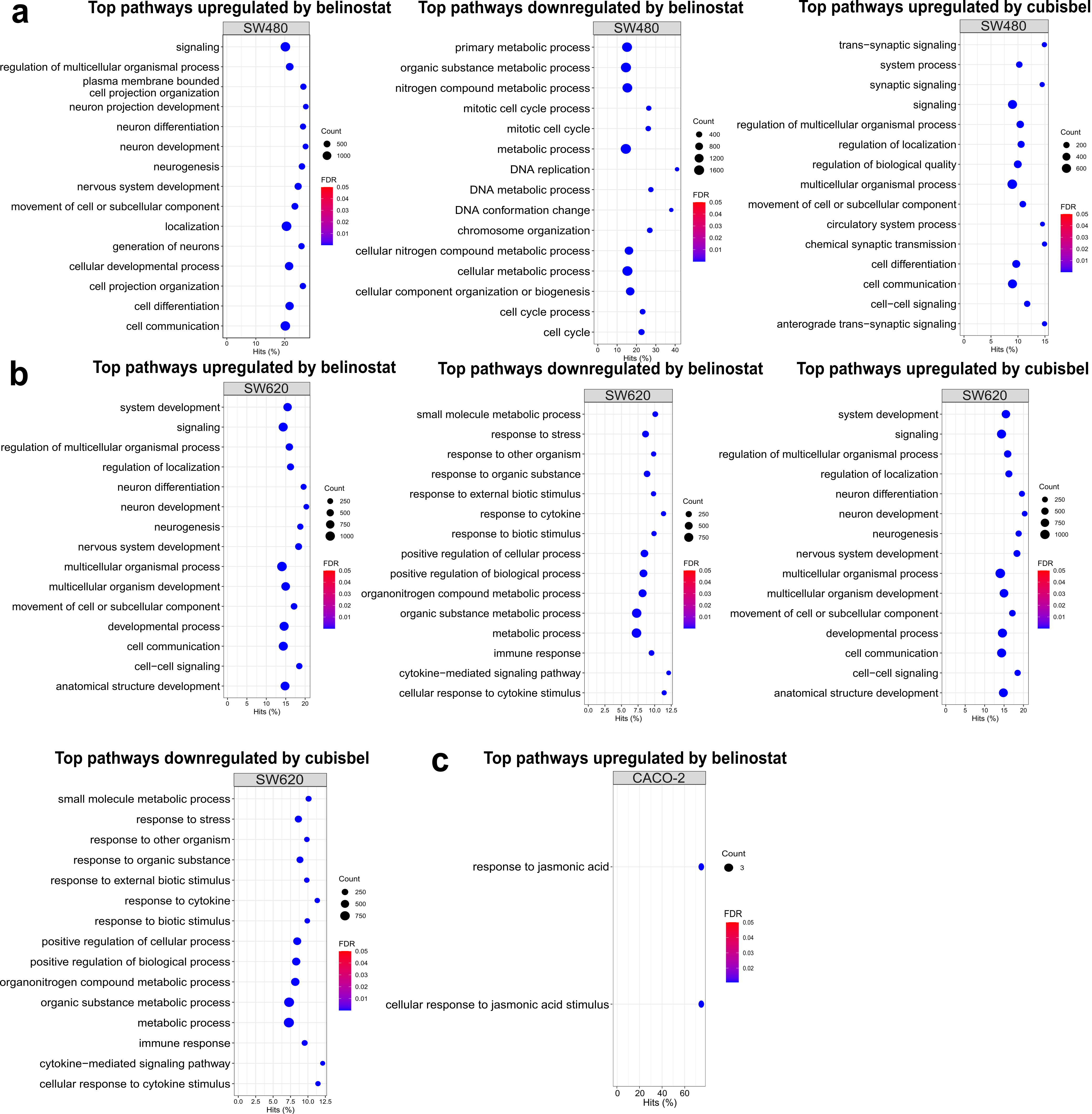


**Supplementary Figure 10. Gene ontology analysis of genes dysregulated by belinostat and Cubisbel in SW480 and SW620 cells.** **(a)** Dot plots of the top 15 GO pathways dysregulated by belinostat and Cubisbel in the SW480 cell line versus DMSO control. The y-axes represents enriched GO categories and x-axes represents percentage of hits or all DEGs belonging to each category. The dots are sized based on gene count and coloured according to their adjusted P-value with a cut-off threshold of p<0.05. No significantly downregulated pathways by Cubisbel were found. **(b)** Dot plots of the top 15 GO pathways dysregulated by belinostat and Cubisbel in the SW620 cell line versus DMSO control. **(c)** Dot plots of the pathways upregulated by belinostat in the CACO-2 cell line versus DMSO control. No pathways significantly downregulated by belinostat or upregulated by Cubisbel were found.


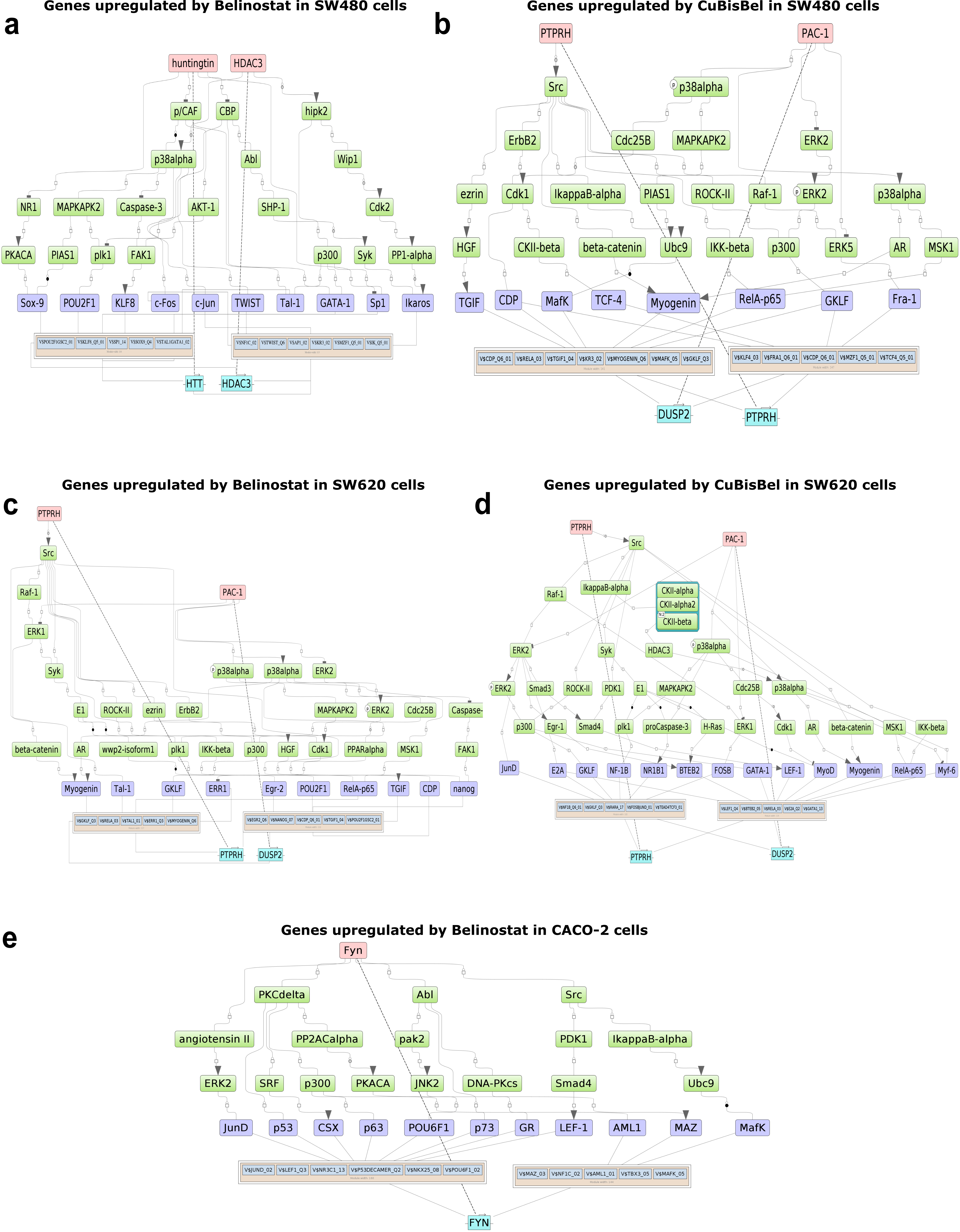


**Supplementary Figure 11. Identification of intracellular regulatory signalling pathways and MRs of genes upregulated by belinostat and Cubisbel in colon cancer cells.** Intracellular signalling networks for genes upregulated by **(a)** belinostat and **(b)** Cubisbel in SW480 cells, networks of genes upregulated by **(c)** belinostat and **(d)** Cubisbel in SW620 cells and genes upregulated by **(e)** belinostat in CACO-2 cells, identified through *in silico* analysis of DEGs. Master regulatory molecules are indicated by pink rectangles, intermediate molecules are green rectangles and transcription factors are indicated by purple rectangles. Enriched binding motifs are highlighted by a green/orange border while example genes from the input list are shown in blue.


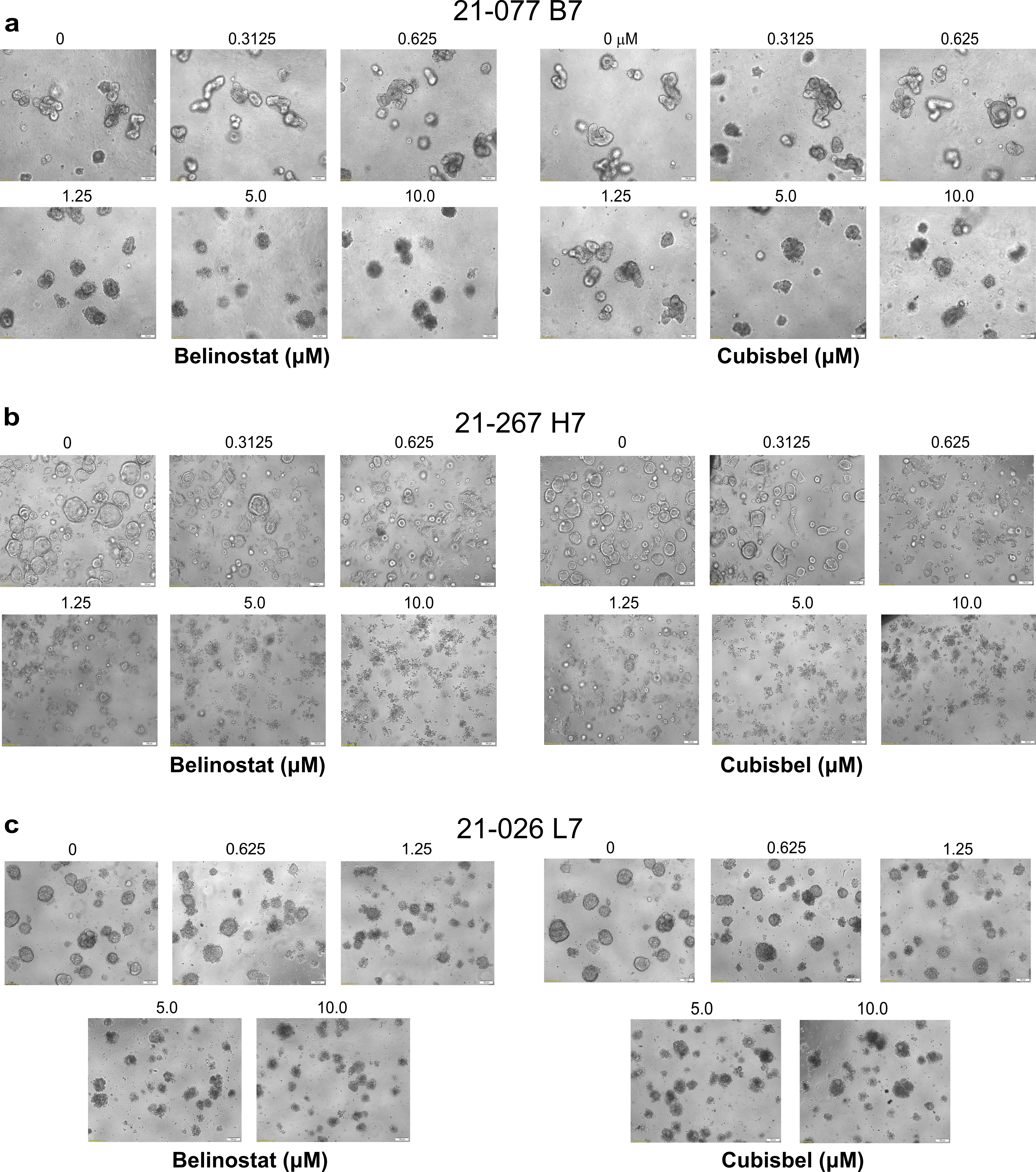


**Supplementary Figure 12. Assessment of the effects of belinostat and Cubisbel on colon cancer PDTO viability.** Representative images of **(a)** 21-077 B7, **(b**) 21-267 H7 and **(c)** 21-026 L7 PDTOs 72 h post-treatment with increasing concentrations of either HDACi. Scale bar = 100 μm.


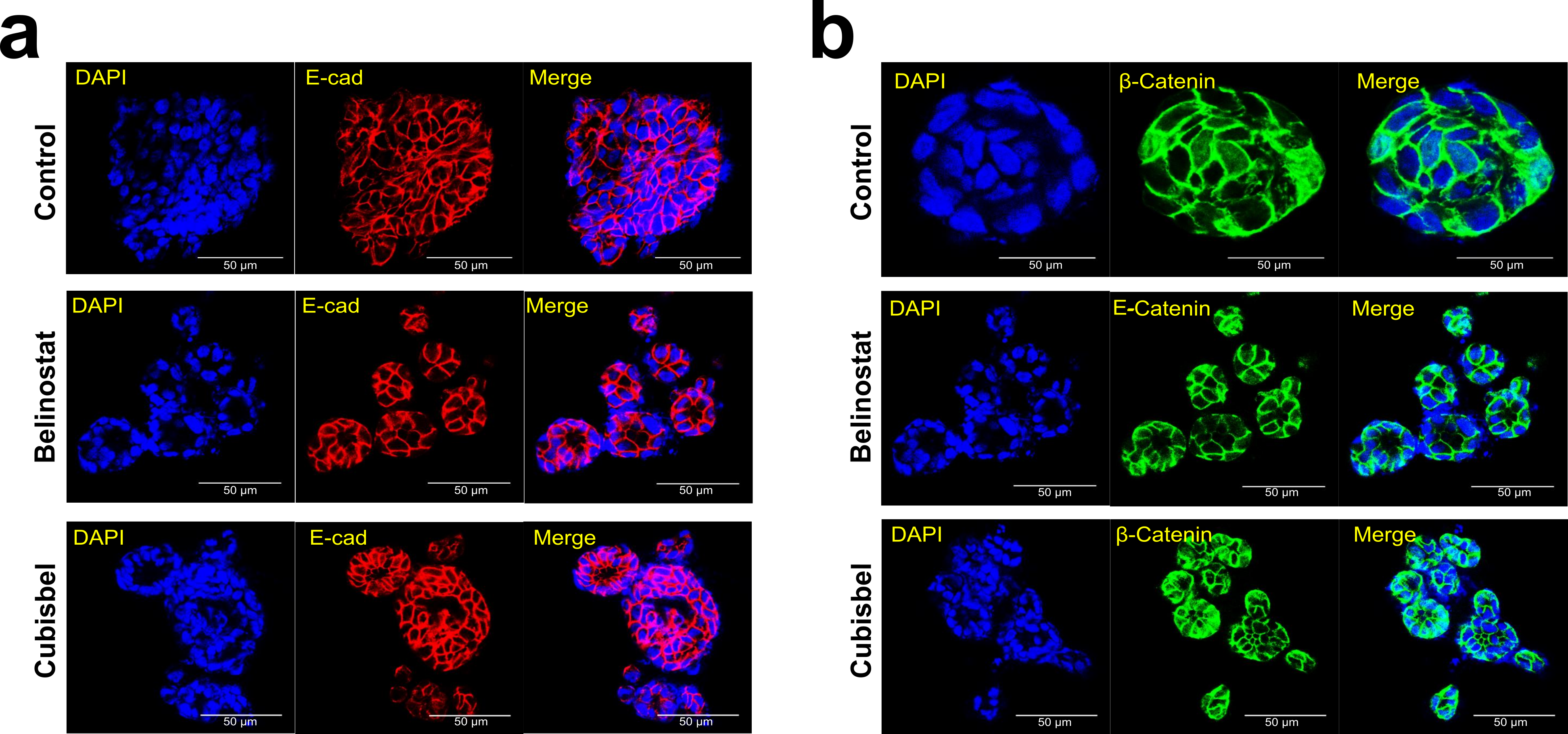


**Supplementary Figure 13. Immunostaining of 3D colon cancer PDTO 21-267 H7 for β-Catenin and E-cadherin following treatment with IC_50_ concentrations of belinostat and Cubisbel**. Representative images were captured using confocal microscopy. Scale bar = 50 μm.


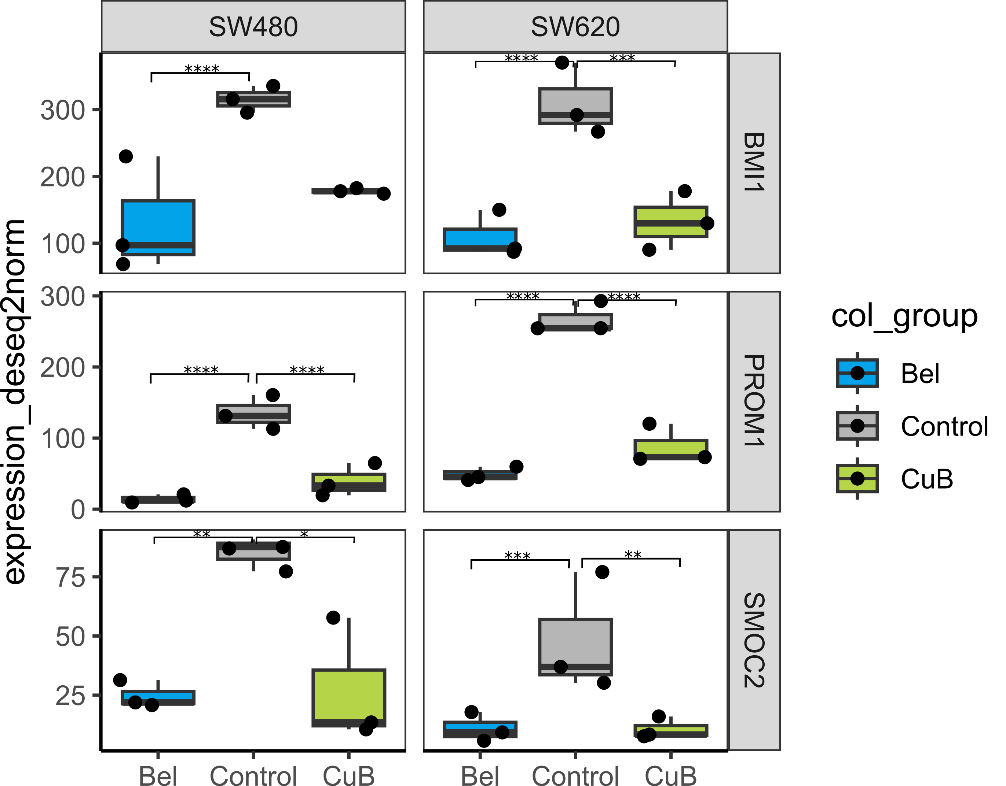


**Supplementary Figure 14. Validation of stem cell marker downregulation in SW480 and SW620 cell lines.** Boxplots showing normalized expression counts of stem cell marker genes downregulated by Cubisbel and belinostat also identified in the colon cancer PDTO. Counts represent the mean of n=3 biological replicates from RNA-Seq data.

**Supplementary Table 3. All significantly differentially expressed genes in SW480, SW620 and CACO-2 cells treated with belinostat or Cubisbel versus DMSO control**

**Supplementary Table 4. Gene ontology pathways significantly dysregulated by belinostat and Cubisbel in colon cancer cells.**

**Supplementary Table 5. Enriched transcription factors and corresponding binding motifs present in promoters of genes dysregulated by belinostat and Cubisbel in colon cancer cells.**

**Supplementary Table 6 Master regulators of genes dysregulated by belinostat and Cubisbel in colon cancer cells.**

**Supplementary Methods**

**Copper-bis-belinostat Synthesis**

[Cu(Bel_-H_)_2_] (Cubisbel) was synthesized by dissolving CuCl_2_.2H_2_O (50 mg, 0.293 mmol) in a minimum volume of water (6 mL). A hot solution of belinostat (186.7 mg, 0.586 mmol) in MeOH (10 mL) was then added. Afterwards, a solution of KOH (32.87 mg, 0.586 mmol) in a minimum volume of water (2 ml) was added to the reaction mixture and was left stirring at room temperature for 45 minutes (min), after which the filtrate was left to slowly evaporate at RT for ~3 days. A green solid was filtered off, washed with cold water, cold MeOH and dried under vacuum (95.66 mg, 0.137 mmol). Characteristic bands for both the carbonyl C=O stretching and C−N−O bending for the hydroxamate functionality in the IR spectrum of the complex showed low and high frequency shifts compared to the free ligand, respectively These observations were in the agreement with a bidentate *O,O′*-chelating mode of hydroxamates to a Cu(II) metal centre^(22)^. IR selected bands (cm^-1^, ATR): 3241, 3036, 1641, 1580, 1525, 1475, 1418, 1333, 1153, 1067, 1019, 972, 791. Anal. Calcd for C_30_H_26_CuN_4_O_8_S_2_: C, 51.61; H, 3.75; N, 8.02; Cu, 9.10. Found: C, 51.55; H, 3.74; N, 7.85; Cu, 9.31.

**Complex purification and analysis**

All commercially available reagents were purchased from Sigma Aldrich (Arklow, Ireland) without further purification. Infrared spectra were recorded on a Nicolet iS10 FT-IR (Thermo Fisher Scientific, Waltham, Massachusetts, USA) and analysed using OMNIC software (version 9.6, Thermo Fisher Scientific). Frequencies (ν) are quoted in cm^-1^. ESI mass spectra were acquired using a Bruker micrOTOF-Q III spectrometer interfaced to a Dionex UltiMate 3000 LC in positive and negative modes as required. The instrument was calibrated using a tune mix solution, (Agilent Technologies ESI-l Low concentration tuning mix) and also used as an internal lock mass. Masses were recorded over the range 100-1400 *m/z*. Operating conditions were as follows: end-plate offset 500 V capillary 4500 V, nebulizer 2.0 Bar, dry gas 8.0 l/min, and dry temperature 180 ˚C. MicroTof control 3.2 and HyStar 3.2 software were used to carry out the analysis. Mass results are quoted in *m/z*. Elemental analysis experiments were conducted in the School of Chemistry, University College Dublin. Elemental analysis (C, H, N) was carried out on an Exeter Analytical CE440 elemental analyzer. Cu analysis was carried out on a Varian 55B atomic absorption spectrometer. Stability analysis of Cubisbel in 100% DMSO was carried out at various timepoints ranging from 0 – 10 days. At each timepoint, ultraviolet-visible (UV-Vis) spectra were recorded on the SHIMADZU spectrophotometer (model 1900i) at 400 – 800 nm. Generated UV-Vis data were analyzed using UVProbe (v 2.70).

**Standard and sample preparation and analysis by LC-MS**

In preparation for LC-MS analysis, stock solution of belinostat, belinostat glucuronide and an internal standard (malic acid-d3) were prepared independently at 10 mM, 2 mM, and 0.7 mM, respectively, and stored at -20 °C for calibration. On the day of the assay, solutions were serially diluted in methanol to obtain the lower calibration working solutions. The final analyte concentrations for the belinostat standard curve were 54.69, 27.34, 13.67, 6.83, 1.37, 0.27, and 0.05 μM. The final analyte concentrations for belinostat glucuronide standard curve were 54.69, 27.34, 13.67, 6.83, 1.37, 0.27, 0.05 and 0.01 μM.

HLM samples were centrifuged for 5 min at 4 ^o^C at 2750 x *g* and the supernatant was diluted 10 times. 70 μL of diluted supernatant was mixed with 10 μL of the internal standard and analyzed by LC-MS.

The samples were analysed by UPLC-QTOF-MS (Agilent Technologies, Santa Clara, CA, USA), which consisted of 1290 Infinity II LC system and an Agilent Jetstream (AJS) electrospray ionization (ESI) source coupled to a 6545 QTOF mass spectrometer. The chromatography was performed in reverse phase mode using Zorbax eclipse plus C18 (2.1 × 50 mm, 1.8 µm) column.

The LC parameters used for the analysis were: Column temperature, 40 °C; Injection volume, 3 µl; Flow rate, 0.50 ml min^−1^; Mobile phase, 0.1% formic acid in water (Eluent A) and 0.1% formic acid in acetonitrile (Eluent B); Gradient conditions, 10%B (0-3.8 min), 50%B (3.8-3.85 min), 90%B (3.85-4.0 min), 90%B (4-5.5 min), 10%B(5.5-5.6 min), 10%B (5.6-7 min). The MS parameters used for the analysis were: drying gas temperature, 325 °C; drying gas flow rate, 10 l min^−1^; sheath gas temperature, 350°C; sheath gas flow rate, 11 l min^−1^; nebulizer pressure, 45 psi; capillary voltage, 3500 V; nozzle voltage, 1000 V; fragmentor voltage, 100 V; skimmer, 45 V. An analysis mass range of *m/z* 50–600. The negative ionisation mode was selected for analysis.

Data were acquired using MassHunter acquisition B.08.00 software (B.08.00.8058.3 Sp1 Agilent Technologies) and processed using MassHunter Qualitative Analysis (B.07.00 Sp2 Agilent Technologies) software. Belinostat and belinsotat glucuronide were quantified based on the standard curve.
