# Supplementary material for: Complexation of histone deacetylase inhibitor belinostat to Cu(II) prevents premature metabolic inactivation in vitro and demonstrates potent anti-cancer activity in vitro and ex vivo in colon cancer
Source: Cell Oncol (Dordr). 2023 Nov 7;47(2):533–53. doi: 10.1007/s13402-023-00882-x (PMC11090832; doi:10.1007/s13402-023-00882-x)
Supplement: Supplementary file 2 — Supplementary file2 (DOCX 26 KB) [file 13402_2023_882_MOESM2_ESM.docx]

| Gene | Forward | Reverse |
| --- | --- | --- |
| CD22 | GCACCCTGAAACCCTCTACG | ATCAAACTTCGAGGTGTTCTTGT |
| ZNF114 | GATTGGGCAACTCCATGTAAAAC | CTGATGCTCGTGAGACACACT |
| LGALSL | AGTTCAAGCGGACGTGTACTT | GGTTGAGGTCTACGATGCCC |
| GNG2 | ATGGAAGCCAATATCGACAGGATA | CTTCTCCCTAAACGGGTTTTCTG |
| NTRK2 | TCGTGGCATTTCCGAGATTGG | TCGTCAGTTTGTTTCGGGTAAA |

**Supplementary Table 1. RT-qPCR primer sequences**
