# Supplementary material for: Complexation of histone deacetylase inhibitor belinostat to Cu(II) prevents premature metabolic inactivation in vitro and demonstrates potent anti-cancer activity in vitro and ex vivo in colon cancer
Source: Cell Oncol (Dordr). 2023 Nov 7;47(2):533–53. doi: 10.1007/s13402-023-00882-x (PMC11090832; doi:10.1007/s13402-023-00882-x)
Supplement: Supplementary file 3 — Supplementary file3 (DOCX 22 KB) [file 13402_2023_882_MOESM3_ESM.docx]

|  | *21-077 B7* | *21-267 H7* | *21-026 L7* |
| --- | --- | --- | --- |
| **Adenocarcinoma location** | Hepatic flexure | Cecum | Sigmoid |
| **Stage** | II | II | II |
| **Grade** | Moderately differentiated | Poorly differentiated | Poorly differentiated |
| **Gender** | Male | Male | Female |
| **Ethnicity** | Caucasian | Caucasian | Caucasian |
| **Age at surgery** | 73 | 82 | 71 |
| **Smoking history** | Ex-smoker | Never smoked | Smoker (30 years) |
| **Family history of other cancers** | yes | no | yes |

**Supplementary Table 5. Clinical and pathological features of the colon cancer PDTOs.**
